# Supplementary material for: Molecular Constraints on Synaptic Tagging and Maintenance of Long-Term Potentiation: A Predictive Model
Source: PLoS Comput Biol. 2012 Aug 2;8(8):e1002620. doi: 10.1371/journal.pcbi.1002620 (PMC3410876; doi:10.1371/journal.pcbi.1002620)
Supplement: Text S1 — A compilation of commented Java programs that execute the simulations presented in Figs. 2–8 of the manuscript. Each program should be saved to a separate file the name of which matches the class name of the program, e.g., the first program in Text S1 should be saved as a file denoted “basicmod.java”. These files can then be immediately compiled and executed. (RTF) [file pcbi.1002620.s001.rtf]

Supporting Java Programs for Smolen, Baxter, and Byrne; “Molecular Constraints on Synaptic Tagging and Maintenance of Long-Term Potentiation: A Predictive Model”.
This text file contains seven JAVA programs that execute the simulations depicted in Figures 3-9 of the manuscript. The programs are separated in the text file by horizontal lines, and each program begins with the line “import java.io.*;”. 
In order, these seven programs are:  1) basicmod.java, which executes the simulations of Figs. 3A-C, 4, 5, 6A1,6A2, 7, and 8.  2) curv.java, which generates the traces of Fig. 3D.  3) winltp.java, which generates the sloped LTP window in Fig. 6B1.  4) wsltp.java, which generates the square LTP window in Fig. 6B1.  5) windep.java, which generates the LTD window in Fig. 6B2.  6) stochpkm.java, which executes the simulations of Figs. 9A-B.  7) elemstoch.java, which executes the simulation of Fig. 9C.
Two notes when breaking out individual programs for use.   1) When saving each program to an individual file, make sure there are no blank lines above the “import…” line to avoid a compile error.  2) The file name must match the class name in the second line of the program. For example, the first program below has “public class basicmod”, and should be saved as basicmod.java. 


import java.io.*;

public class basicmod

/* Simulates basic LTP and LTD protocols (Figs. 3 and 5 in PKM manuscript), effects of inhibitors (Fig. 4), and tagging and crosstagging experiments (Figure 6 top panels, Figure 7, and Figure 8). */

 {  public static void main (String args[]) throws IOException {

    PrintWriter out1 = new PrintWriter(new FileWriter("casyn.txt"));
    PrintWriter out2 = new PrintWriter(new FileWriter("ck2act.txt"));
    PrintWriter out3 = new PrintWriter(new FileWriter("erkact.txt"));
    PrintWriter out4 = new PrintWriter(new FileWriter("pp.txt"));
    PrintWriter out5 = new PrintWriter(new FileWriter("tagp1.txt"));
    PrintWriter out6 = new PrintWriter(new FileWriter("tagd1.txt"));
    PrintWriter out7 = new PrintWriter(new FileWriter("tagd2.txt"));
    PrintWriter out8 = new PrintWriter(new FileWriter("tltp.txt"));
    PrintWriter out9 = new PrintWriter(new FileWriter("tltd.txt"));
    PrintWriter out10 = new PrintWriter(new FileWriter("cadend.txt"));
    PrintWriter out11 = new PrintWriter(new FileWriter("ckdact.txt"));
    PrintWriter out12 = new PrintWriter(new FileWriter("erkdact.txt"));
    PrintWriter out13 = new PrintWriter(new FileWriter("psit1.txt"));
    PrintWriter out14 = new PrintWriter(new FileWriter("psit2.txt"));
    PrintWriter out15 = new PrintWriter(new FileWriter("prp.txt"));
    PrintWriter out16 = new PrintWriter(new FileWriter("pkmd.txt"));
    PrintWriter out17 = new PrintWriter(new FileWriter("pkmsn.txt"));
    PrintWriter out18 = new PrintWriter(new FileWriter("fsyn.txt"));
    PrintWriter out19 = new PrintWriter(new FileWriter("nsyn.txt"));
    PrintWriter out20 = new PrintWriter(new FileWriter("wsyn.txt"));
    PrintWriter out21 = new PrintWriter(new FileWriter("kfraf.txt"));
    PrintWriter out22 = new PrintWriter(new FileWriter("kfrafd.txt"));

/* Following are adjustable parameters for timesteps and times to start and stop output and deliver stimuli.*/

    double dt=0.0002; // high-res timestep (min)
    double delta=0.01; // low-res timestep (min)

    double recstart=1940.0; // Time to start writing data
    double stime=2000.0; // Time of stimulus onset

    double isi=5.0;      // Spacing between tetanic stimuli

//Time to end simulation and stop writing data

	double recend=2360.0;

     int recintvl=10; // Intervals to record, in units of the low-res timestep delta

    double tref; /* Time since stimulus onset at Synapse 1. If this is a tagging simulation, synapse 2 will also be stimulated, in which case a second variable, tref2, marks time since stimulus at synapse 2. */

/* Time offset for crosstagging expts. Defined as (time of strong at S2 for PRP's) – (time of weak at S1 for tag). So positive doff is weak before strong. tref2 is then tref – doff. */

     double doff=20.0;
     double tref2;

//PROGRAM VARIABLES

// CAMKII
	double ck2act;

// SYNAPTIC ERK MAPK CASCADE. START WITH INTERMEDIATE VARIABLES NEEDED TO SIMULATE DYNAMIC ACTIVATION PROFILE OF RAF
	double kfraf;
	double kfrafd;
	double f1up;
	double f1dn;
	double f2up;
	double f2dn;
	double f3up;
	double f3dn;
	double g1up;
	double g1dn;
	double g2up;
	double g2dn;
	double g3up;
	double g3dn;
	double g4up;
	double g4dn;
	double g5up;
	double g5dn;
	double g6up;
	double g6dn;

// ERK AND UPSTREAM ENZYMES
	double raf;
	double rafp;
	double mkk;
	double mkkp;
	double mkkpp;
	double erk;
	double erkp;
	double erkpp;
	double erkact;

// SYNAPTIC LTP TAG VARIABLES. tagp1 is the site that needs to be phosphorylated by CAMKII to set the tag.
	double tagp1;
	double tltp;

/* SYNAPTIC LTD TAG VARIABLES. tagd1 is the site that needs to be phosphorylated by ERK, tagd2 is the site that needs to be dephosphorylated by PP. */
	double tagd1;
	double tagd2;
	double tltd;
	double ppact; // LTD phosphatase activity. Activate similar to CAMKII but with lower KD for casyn.
	
/* PRP, PKM variables. psit1, 2 are the phosphorylation sites to allow synthesis of the plasticity-related protein, “prp”, needed to drive LTD. */
	double psit1;
	double psit2;
	double prp;

	double pkmd; // dendritic and synaptic PKM
	double pkmsn;

/* SYNAPTIC WEIGHT VARIABLES. Fsyn and nsyn are respectively saturation of AMPA-R docking sites and number of docking sites. nsyn is modeled as real instead of integer to allow for a differential-equation based model of its dynamics */
	double wsyn;
	double fsyn;
	double nsyn;

/* Duplicate variables for the dendritic region – CaM kinase and ERK cascade. These variables are activated by strong stimuli to synapse 1(standard LTP or LTD) or synapse 2 (STC or SCTC protocols). */

	double ckdact;
	double erkdact;
	double rafd;
	double rafp2;
	double mkk2;
	double mkkp2;
	double mkkpp2;
	double erk2;
	double erkp2;
	double erkpp2;

// TIME DERIVATIVES

    	double dv1dt;
    	double dv2dt;
    	double dv3dt;
    	double dv4dt;
    	double dv5dt;
    	double dv6dt;
    	double dv7dt;
    	double dv8dt;
    	double dv9dt;
    	double dv10dt;
    	double dv11dt;
    	double dv12dt;
    	double dv13dt;
    	double dv14dt;
	double dv15dt;
	double dv16dt;
	double dv17dt;
	double dv18dt;
	double dv19dt;
	double dv20dt;
	double dv21dt;
	double dv22dt;
	double dv23dt;
	double dv24dt;
	double dv25dt;
	double dv26dt;

// Synaptic and dendritic Ca concentrations and powers
	double casyn;
	double cadend;
	double powca; // casyn, synapse 1
	double powcad; // cadend, Ca pool for PRP synthesis
	double powkc; // for camkii activation
	double powkc2; // for pp (pp) activation
	double powkcd; // Dendritic “camk” activation for PKM

// WORK IN CONCENTRATION UNITS OF uM, TIME UNITS OF MIN

// MODEL PARAMETERS FOLLOW:

/* BASAL CA, STIMULUS AMPLITUDES. These are for LTP protocols. */

	double Cabas=0.04;

/* FIRST GROUP OF AMPLITUDES ARE FOR TAG SETTING. THEY ARE AT SYNAPSE 1. */

// Calcium levels and maximal amplitudes of Raf activation, at synapse 1, in response to tetanus or chemical stimulus
	double AMPTETCA=1.4;
	double AMPCHEMCA=0.24;

	double AMPTETSTIM=0.006;
	double AMPCHEMSTIM=0.007; 

// Calcium levels and maximal amplitudes of Raf activation, at synapse 1, in response to SLFS or WLFS

	double AMPSLCA=0.17;
	double AMPSLST=0.02;
	double AMPWLCA=0.16;
	double AMPWLST=0.02;

/* Second group of amplitudes, for dendritic calcium amplitudes and maximal amplitudes of dendritic Raf activation. */

	double AMPTETCAD=0.65;
	double AMPCHEMCAD=0.24;

	double AMPTETSTIMD=0.03;
	double AMPCHEMSTIMD=0.007;

/* Dendritic calcium and Raf activation amplitudes during SLFS and WLFS. WLFS does not elevate Ca above basal. */

	double AMPSLCAD=0.17;
	double AMPSLSTD=0.017;
	double AMPWLCAD=0.04;
	double AMPWLSTD=0.006;

// Rate constants and Ca dissociation constant for CAMKII 
	double kfck2=200.0;
	double tauck2=1.0;
	double Kck2=1.4;

/* LOWER BINDING CONSTANT FOR “DENDRITIC” CAMKII THAT DRIVES PKM SYNTHESIS. CHOSEN LOWER SO THAT PKM CAN BE SYNTHESIZED BY SLFS */

	double Kck2d=0.6;

// Rate constants and Ca dissociation constant for LTD phosphatase
	double kfpp=2.0;
	double taupp=2.0;
	double Kpp=0.225;

// Conserved amounts of Raf, Mek, ERK.
	double raftot=0.25;
	double mkktot=0.25;
	double erktot=0.25;

// ERK pathway rate and Michaelis constants
	double kbraf=0.12;
	double kfmkk=0.6;
	double kbmkk=0.025;
	double Kmkk=0.25;
	double kferk=0.52;
	double kberk=0.025;
	double Kmk=0.25;
	double kfbasraf=0.003;
	double kfbasrafd=0.003;
	double taufras=0.5;
	double tausras=4.0;

// Rate constants for the phosphorylation needed for LTP synaptic tagging

	double kphos1=0.45;
	double kdeph1=0.006;

// Rate constants for the phosphorylation and dephosphorylation needed for LTD synaptic tagging.

	double kphos4=2.0;
	double kdeph4=0.011;
	double kphos5=0.011;
	double kdeph5=0.04;

/* Rate constants for the phosphorylations needed for synthesis of PRP, the LTD protein. */
	double kphos7=4.0;
	double kdeph7=0.1;
	double kphos8=0.015;
	double kdeph8=0.02;

// Maximal and basal transcription rates for PRP, decay of PRP
	double ktrans1=2.2;
	double vtrbas1=0.001;
	double tauprp=45.0;

// PKM parameters
	double kpkmon=0.5;
	double kpkmon2=0.055;
	double taupkm=50.0;
	double vtrbaspkm=0.0003;
	double Kpkm = 0.75;
	double kexpkm=0.0025;
	double klkpkm=0.012;
	double Vsd=0.03;

// Inhibition factors for enzymes and PRP's. Can be used to model effects of inhibition of PKM, CAMKII, ERK, or protein synthesis on LTP and LTD.

	double inherk;
	double inhmkk;
	double inhck2;
	double inhprot;
	double inhprp;
	double inhpp;
	double inhpkm;

// Rate constants and other parameters for synaptic 
// weight changes, that is, changes in variables Fsyn, Nsyn, Wsyn 

	double kltp=0.014;
	double kltd=0.03;
	double tfsyn=30.0;
	double tnsyn=600.0;
	double vfbas=0.01;
	double vdbas=0.0033;


// TIME STUFF, COUNTERS, VALUE ARRAY

    double time=0.0; // time (minutes)
    double timewrite;

    int i, k, j, l; // counters

    double[] values=new double[28];

// VARIABLE INITIALIZATION. Use small but nonzero initial values
// to avoid extremely small numbers in output files.

	values[1]=0.001;
	values[2]=0.5*raftot;
	values[3]=0.3*mkktot;
	values[4]=0.4*mkktot;
	values[5]=0.3*erktot;
	values[6]=0.4*erktot;
	values[7]=0.001;
	values[8]=0.001;
	values[9]=0.001;
	values[10]=0.001;
	values[11]=0.001;
	values[12]=0.5*raftot;
	values[13]=0.3*mkktot;
	values[14]=0.4*mkktot;
	values[15]=0.3*erktot;
	values[16]=0.4*erktot;
	values[17]=0.001;
	values[18]=0.001;
	values[19]=vtrbas1*tauprp;
	values[20]=vtrbaspkm*taupkm;
	values[21]=0.001;
	values[22]=0.01;
	values[23]=0.01;
	values[24]=0.01;
	values[25]=0.01;

// MAIN LOOP (LARGER TIMESTEP)

        k=1;
        do {  

// INNER SIMULATION LOOP (Smaller timestep dt)

            j=1;
            do {

		tref=time-stime;
		casyn=Cabas;
		cadend=Cabas;

		ck2act=values[1];

		raf=values[2];
		mkk=values[3];
		mkkpp=values[4];
		erk=values[5];
		erkpp=values[6];

		ppact=values[7];

		tagp1=values[8];

		tagd1=values[9];
		tagd2=values[10];

		ckdact=values[11];

		rafd=values[12];
		mkk2=values[13];
		mkkpp2=values[14];
		erk2=values[15];
		erkpp2=values[16];

		psit1=values[17];
		psit2=values[18];

		prp=values[19];

		pkmd=values[20];

		pkmsn=values[21];

		fsyn=values[22];

		nsyn=values[23];

		wsyn=values[24];

/* Synaptic variables are simulated at Synapse 1. */

       f1up=0.0;
       f1dn=1.0;
       f2up=0.0;
       f2dn=1.0;
       f3up=0.0;
       f3dn=1.0;
       g1up=0.0;
       g1dn=1.0;
       g2up=0.0;
       g2dn=1.0;
       g3up=0.0;
       g3dn=1.0;
       g4up=0.0;
       g4dn=1.0;
       g5up=0.0;
       g5dn=1.0;
       g6up=0.0;
       g6dn=1.0;
       kfraf=kfbasraf;
       kfrafd=kfbasrafd;

// Tetanic protocol. For submission of basicmod, only this protocol is uncommented. The other protocols are commented out (bracketed with comment marks).

		if (tref > 0.0 && tref < 0.05)
		  {
		  casyn=AMPTETCA;
		  cadend=AMPTETCAD;
		  }
		if (tref > (0.0+isi) && tref < (0.0+isi+0.05))
		  {
		  casyn=AMPTETCA;
		  cadend=AMPTETCAD;
		  }
		if (tref > (0.0+2.0*isi) && tref < (0.0+2.0*isi+0.05))
		  {
		  casyn=AMPTETCA;
		  cadend=AMPTETCAD;
		  }
// NOTE, REMOVE THIS TEXT PARAGRAPH WHEN COMPILING AND RUNNING PROGRAM
/* The following rather complex code is needed to simulate a dynamic profile of Raf activation. It sets time-dependent variables, f1up etc., at each tetanus. These variables will later be used to generate a multi-peaked time course for kfraf, the rate constant for Raf activation. That time course corresponds to an empirical time course of Ras activity, as discussed in the PKM manuscript. */

		if (tref > 0.0 && tref < 60.0)
		  {
		  f1up = 1.0-Math.exp(-(tref)/taufras);
		  f1dn = (AMPTETSTIM-kfbasraf)* Math.exp(-(tref)/tausras);
		  }
		if (tref > (0.0+isi) && tref < 60.0)
		  {
		  f2up = 1.0-Math.exp(-(tref-isi)/taufras);
		  f2dn = (AMPTETSTIM-kfbasraf)* Math.exp(-(tref-isi)/tausras);
		  }
		if (tref > (0.0+2.0*isi) && tref < 60.0)
		  {
		  f3up = 1.0-Math.exp(-(tref-2.0*isi)/taufras);
		  f3dn = (AMPTETSTIM-kfbasraf)* Math.exp(-(tref-2.0*isi)/tausras);
		  }
		if (tref > 0.0 && tref < 60.0)
		  {
		  g1up = 1.0-Math.exp(-(tref)/taufras);
		  g1dn = (AMPTETSTIMD-kfbasrafd)* Math.exp(-(tref)/tausras);
		  }
		if (tref > (0.0+isi) && tref < 60.0)
		  {
		  g2up = 1.0-Math.exp(-(tref-isi)/taufras);
		  g2dn = (AMPTETSTIMD-kfbasrafd)* Math.exp(-(tref-isi)/tausras);
		  }
		if (tref > (0.0+2.0*isi) && tref < 60.0)
		  {
		  g3up = 1.0-Math.exp(-(tref-2.0*isi)/taufras);
		  g3dn = (AMPTETSTIMD-kfbasrafd)* Math.exp(-(tref-2.0*isi)/tausras);
		  }


// Chem LTP
// Activates ERK
// Assume Chem treatment elevates Ca, otherwise no significant LTP.

/*
		if (tref > 0.0 && tref < 30.0)
		  {
		  casyn=AMPCHEMCA;
		  cadend=AMPCHEMCAD;
		  }
		if (tref > 0.0 && tref < 60.0)
		  {
		  f1up= (AMPCHEMSTIM-kfbasraf)*(1.0-Math.exp(-(tref)/taufras));
		  if (tref > 30.0) {f1dn=Math.exp(-(tref-30.0)/tausras);}
		  }
		if (tref > 0.0 && tref < 60.0)
		  {
		  g1up= (AMPCHEMSTIMD-kfbasrafd)*(1.0-Math.exp(-(tref)/taufras));
		  if (tref > 30.0) {g1dn=Math.exp(-(tref-30.0)/tausras);}
		  }
*/

// WTET PROTOCOL
/*
		if (tref > 0.0 && tref < 0.05)
		  {
		  casyn=AMPTETCA;
		  cadend=AMPTETCAD;
		  }
		if (tref > 0.0 && tref < 60.0)
		  {
		  f1up = 1.0-Math.exp(-(tref)/taufras);
		  f1dn = (AMPTETSTIM-kfbasraf)* Math.exp(-(tref)/tausras);
		  }
		if (tref > 0.0 && tref < 60.0)
		  {
		  g1up = 1.0-Math.exp(-(tref)/taufras);
		  g1dn = (AMPTETSTIMD-kfbasrafd)* Math.exp(-(tref)/tausras);
		  }
*/
// SLFS STIMULUS TO PRODUCE L-LTD
/*
		if ((tref > 0.0) && (tref < 15.0))
		{
			 casyn=AMPSLCA;
			 cadend=AMPSLCAD;
		}
		if (tref > 0.0 && tref < 60.0)
		  {
		  f1up= (AMPSLST-kfbasraf)*(1.0-Math.exp(-(tref)/taufras));
		  if (tref > 15.0) {f1dn=Math.exp(-(tref-15.0)/tausras);}
		  }
		if (tref > 0.0 && tref < 60.0)
		  {
		  g1up= (AMPSLSTD-kfbasrafd)*(1.0-Math.exp(-(tref)/taufras));
		  if (tref > 15.0) {g1dn=Math.exp(-(tref-15.0)/tausras);}
		  }
*/
// WLFS STIMULUS
/*
		if ((tref > 0.0) && (tref < 15.0))
		{
			 casyn=AMPWLCA;
			 cadend=AMPWLCAD;
		}
		if (tref > 0.0 && tref < 60.0)
		  {
		  f1up= (AMPWLST-kfbasraf)*(1.0-Math.exp(-(tref)/taufras));
		  if (tref > 15.0) {f1dn=Math.exp(-(tref-15.0)/tausras);}
		  }
		if (tref > 0.0 && tref < 60.0)
		  {
		  g1up= (AMPWLSTD-kfbasrafd)*(1.0-Math.exp(-(tref)/taufras));
		  if (tref > 15.0) {g1dn=Math.exp(-(tref-15.0)/tausras);}
		  }
*/


/* NOW give protocols for STC or SCTC. Synapse 2, not explicitly modeled, is strongly stimulated by STET or SLFS. Dendritic Ca is elevated and dendritic ERK is activated. */

	tref2 = tref-doff; // Offsets Synapse 2 stimulus to be doff minutes later than Tag stimulus at synapse 1.

// Tetanic protocol
/*
		if (tref2 > 0.0 && tref2 < 0.05)
		  {
		  cadend=AMPTETCAD;
		  }
		if (tref2 > (0.0+isi) && tref2 < (0.0+isi+0.05))
		  {
		  cadend=AMPTETCAD;
		  }
		if (tref2 > (0.0+2.0*isi) && tref2 < (0.0+2.0*isi+0.05))
		  {
		  cadend=AMPTETCAD;
		  }
		if (tref2 > 0.0 && tref2 < 60.0)
		  {
		  g4up = 1.0-Math.exp(-(tref2)/taufras);
		  g4dn = (AMPTETSTIMD-kfbasrafd)* Math.exp(-(tref2)/tausras);
		  }
		if (tref2 > (0.0+isi) && tref2 < 60.0)
		  {
		  g5up = 1.0-Math.exp(-(tref2-isi)/taufras);
		  g5dn = (AMPTETSTIMD-kfbasrafd)* Math.exp(-(tref2-isi)/tausras);
		  }
		if (tref2 > (0.0+2.0*isi) && tref2 < 60.0)
		  {
		  g6up = 1.0-Math.exp(-(tref2-2.0*isi)/taufras);
		  g6dn = (AMPTETSTIMD-kfbasrafd)* Math.exp(-(tref2-2.0*isi)/tausras);
		  }
*/

// SLFS STIMULUS TO PRODUCE L-LTD
/*
		if (tref2 > 0.0 && tref2 < 15.0 && cadend < AMPSLCAD)
		{
			 cadend=AMPSLCAD;
		}
		if (tref2 > 0.0 && tref2 < 60.0)
		  {
	 g4up= (AMPSLSTD-kfbasrafd)*(1.0-Math.exp(-(tref2)/taufras));
		  if (tref2 > 15.0) {g4dn=Math.exp(-(tref2-15.0)/tausras);}
		  }
*/

/* Only now, after all protocols are given, are the time-dependent factors f1up…, g1up…, combined to give time courses of Raf activation (of kfraf). The interval -300 to 300 covers the range within which, for all protocols, the difference of kfraf from its basal value, kfbasraf (or, for dendrite, kfbasrafd) is not negligible. */
		if (tref > -300.0 && tref < 300.0)
		  {
kfraf=kfbasraf + (f1up*f1dn+f2up*f2dn+f3up*f3dn);
kfrafd=kfbasrafd + (g1up*g1dn+g2up*g2dn+g3up*g3dn) + (g4up*g4dn+g5up*g5dn+g6up*g6dn);
		  }


// Set inhibition factors to 1 (no inhibition) and then allow user to set strength and duration of any inhibitor applications.

		inherk=1.0;
		inhck2=1.0;
		inhprot=1.0;
		inhprp=1.0;
		inhmkk=1.0;
		inhpp=1.0;
		inhpkm=1.0;

		if ((tref > -0.001) && (tref < 20.0))
		  {

//			inherk=0.1; 

//			inhck2=0.1;

//			inhprot=0.5; 

//			inhprp=0.2; 

//			inhmkk=0.2; 

//			inhpp=0.1; 

//			inhpkm=0.2;
		  }

// BEGINNING OF CODE FOR UPDATING DYNAMIC VARIABLES. 

// DEFINE SOME AUXILIARY FUNCTIONS
		powca=casyn*casyn*casyn*casyn;
		powcad=cadend*cadend*cadend*cadend;

		powkc=Kck2*Kck2*Kck2*Kck2;
		powkcd=Kck2d*Kck2d*Kck2d*Kck2d;
		powkc2=Kpp*Kpp*Kpp*Kpp;

		rafp=raftot-raf;
		mkkp=mkktot-mkk-mkkpp;
		erkp=erktot-erk-erkpp;

		rafp2=raftot-rafd;
		mkkp2=mkktot-mkk2-mkkpp2;
		erkp2=erktot-erk2-erkpp2;

// Activation of CAMKII by a power of synaptic calcium, hill function,
// uses fourth powers.

		dv1dt = kfck2*(powca/(powca+powkc)) - ck2act/tauck2;

// ODEs for synaptic ERK cascade

		dv2dt = -kfraf*raf+kbraf*rafp;

dv3dt = -inhmkk*kfmkk*rafp*mkk/(mkk+Kmkk)+kbmkk*mkkp/(mkkp+Kmkk); 

		dv4dt = inhmkk*kfmkk*rafp*mkkp/(mkkp+Kmkk)-kbmkk*mkkpp/(mkkpp+Kmkk);

		dv5dt = -kferk*mkkpp*erk/(erk+Kmk)
			+kberk*erkp/(erkp+Kmk);

		dv6dt = kferk*mkkpp*erkp/(erkp+Kmk)
			 -kberk*erkpp/(erkpp+Kmk);

		erkact = erkpp;

// ODE for LTD phosphatase activation

		dv7dt = kfpp*(powca/(powca+powkc2))-ppact/taupp;

// ODE for LTP tag phosphorylation

		dv8dt = kphos1*inhck2*ck2act*(1.0-tagp1) - kdeph1*tagp1;


// ODEs for phosphorylation and dephosphorylation of LTD tag sites

		dv9dt = inherk*kphos4*erkact*(1.0-tagd1)-kdeph4*tagd1;


		dv10dt = inhpp*kdeph5*ppact*(1.0-tagd2)-kphos5*tagd2;

// As discussed in manuscript, the LTP and LTD tags are given as products of sites
		tltp = tagp1*tagp1;
		tltd = tagd1*tagd2;

/* ODEs for dendrite. */

// Dendritic CaM kinase activation

		dv11dt = kfck2*(powcad/(powcad+powkcd)) - ckdact/tauck2;

// Dendritic ERK cascade

		dv12dt = -kfrafd*rafd+kbraf*rafp2;

dv13dt = -inhmkk*kfmkk*rafp2*mkk2/(mkk2+Kmkk)+kbmkk*mkkp2/(mkkp2+Kmkk);

		dv14dt = inhmkk*kfmkk*rafp2*mkkp2/(mkkp2+Kmkk)-kbmkk*mkkpp2/(mkkpp2+Kmkk);

		dv15dt = -kferk*mkkpp2*erk2/(erk2+Kmk)
			+kberk*erkp2/(erkp2+Kmk);

		dv16dt = kferk*mkkpp2*erkp2/(erkp2+Kmk)
			 -kberk*erkpp2/(erkpp2+Kmk);

		erkdact = erkpp2;

// ODE for a dendritic ERK phosphorylation site needed for both PRP and PKM synthesis

		dv17dt = inherk*kphos7*erkdact*(1.0-psit1)-kdeph7*psit1;

// ODE for phosphorylation by dendritic Cam kinase of a second site that governs PKM synthesis

		dv18dt = kphos8*ckdact*(1.0-psit2)-kdeph8*psit2;

/* ODE for synthesis of the PRP needed for LTD */

		dv19dt = inhprot*inhprp*ktrans1*psit1*psit1-prp/tauprp + inhprot*inhprp*vtrbas1;

/* ODE for dendritic PKM */

		dv20dt = inhprot*kpkmon*psit1*psit2-pkmd/taupkm + inhprot*vtrbaspkm-kexpkm*tltp*pkmd+Vsd*klkpkm*pkmsn;

/* ODE for synaptic PKM */

dv21dt = kexpkm*tltp*pkmd/Vsd-klkpkm*pkmsn + inhprot*vtrbaspkm + kpkmon2*inhpkm*inhpkm*inhprot*pkmsn*pkmsn/(inhpkm*inhpkm*pkmsn*pkmsn+Kpkm*Kpkm)-pkmsn/taupkm;

// Fsyn ODE
		dv22dt = kltp*inhpkm*pkmsn-fsyn/tfsyn + vfbas; 

// nsyn ODE
		dv23dt = -kltd*tltd*prp*nsyn-nsyn/tnsyn + vdbas; 

/* Synaptic weight W is given as the product of fsyn and nsyn, so it doesn't have its own ODE. */
		wsyn=fsyn*nsyn;

		values[1]+=dt*dv1dt;
		values[2]+=dt*dv2dt;
		values[3]+=dt*dv3dt;
		values[4]+=dt*dv4dt;
		values[5]+=dt*dv5dt;
		values[6]+=dt*dv6dt;
		values[7]+=dt*dv7dt;
		values[8]+=dt*dv8dt;
		values[9]+=dt*dv9dt;
		values[10]+=dt*dv10dt;
		values[11]+=dt*dv11dt;
		values[12]+=dt*dv12dt;
		values[13]+=dt*dv13dt;
		values[14]+=dt*dv14dt;
		values[15]+=dt*dv15dt;
		values[16]+=dt*dv16dt;
		values[17]+=dt*dv17dt;
		values[18]+=dt*dv18dt;
		values[19]+=dt*dv19dt;
		values[20]+=dt*dv20dt;
		values[21]+=dt*dv21dt;
		values[22]+=dt*dv22dt;
		values[23]+=dt*dv23dt;
		values[24]=wsyn;


/* Equilibrate basal synaptic weight, over the simulated time prior to LTP or LTP induction, according to basal levels of PKM and PRP. */

		if ((tref < -1.0) && (tref2 < -1.0))
		  {
       fsyn = (kltp*pkmsn+vfbas)/(kltp*pkmsn+1.0/tfsyn);
       values[22] = (kltp*pkmsn+vfbas)/(kltp*pkmsn+1.0/tfsyn);
       
       nsyn = vdbas/(kltd*tltd*prp+1.0/tnsyn);
       values[23] = vdbas/(kltd*tltd*prp+1.0/tnsyn);

		  wsyn = fsyn*nsyn;
		  values[24] = fsyn*nsyn;
		  }

// Increment time
                time=time+dt;

// END INNER LOOP

                j++;
               } while (j <= delta/dt);

// COMPUTE AND PRINT OUTPUT VARIABLES

            if ((time > recstart) && (time < recend) && (k % recintvl == 0))
              {
		timewrite=tref/60.0;

//	OUTPUT CONCENTRATION UNITS WILL BE uM. SCALING FACTORS ARE FOR
//	EASE OF CONCURRENT VISUALIZATION

		   out1.println(timewrite + "\t" + 1.0*casyn);          			   out2.println(timewrite + "\t" + 1.0*ck2act);
               out3.println(timewrite + "\t" + 25.0*erkact);
               out4.println(timewrite + "\t" + 0.1*ppact);
               out5.println(timewrite + "\t" + 1.0*tagp1);
               out6.println(timewrite + "\t" + 1.0*tagd1);
               out7.println(timewrite + "\t" + 1.0*tagd2);
               out8.println(timewrite + "\t" + 1.0*tltp);
               out9.println(timewrite + "\t" + 5.0*tltd);
               out10.println(timewrite + "\t" + 1.0*cadend);
               out11.println(timewrite + "\t" + 0.1*ckdact);
               out12.println(timewrite + "\t" + 25.0*erkdact);
               out13.println(timewrite + "\t" + 1.0*psit1);
               out14.println(timewrite + "\t" + 1.0*psit2);
               out15.println(timewrite + "\t" + 0.1*prp); 
               out16.println(timewrite + "\t" + 2.0*pkmd);
               out17.println(timewrite + "\t" + 1.0*pkmsn);
               out18.println(timewrite + "\t" + 1.0*fsyn);
               out19.println(timewrite + "\t" + 0.5*nsyn);
               out20.println(timewrite + "\t" + 1.0*wsyn);
               out21.println(timewrite + "\t" + 1.0*kfraf);
               out22.println(timewrite + "\t" + 1.0*kfrafd);

              }

            k++;
           } while (k <= recend/delta);

// END OF OUTER LOOP AND OF SIMULATION. CLOSE OUTPUT FILES.

      out1.close();
      out2.close();
	out3.close();
	out4.close();
      out5.close();
	out6.close();
	out7.close();
      out8.close();
	out9.close();
      out10.close();
	out11.close();
	out12.close();
	out13.close();
	out14.close();
	out15.close();
	out16.close();
	out17.close();
	out18.close();
	out19.close();
	out20.close();
	out21.close();
	out22.close();

       }
}


import java.io.*;

public class curv 

/* Program for computing the simple bifurcation curves in the last panel of Figure 3 */

 {  public static void main (String args[]) throws IOException {

    PrintWriter out1 = new PrintWriter(new FileWriter("jj.txt"));

double pkmsn;
double dv21dt;
double value;

double tltp=0.0;
double pkmd=0.0;


// tltp=0.25; // values to eliminate lower state
// pkmd=0.2;


double runn=1.8; // distance on x axis, [pkmsn], for the curve to run

// PKM parameters
	double kpkmon=0.5;
	double kpkmon2=0.055;
	double taupkm=50.0;
	double vtrbaspkm=0.0003;
	double Kpkm = 0.75; // standard Kpkm value is 0.75
	// eliminate upper state Kpkm = 1.0;
	// eliminate lower state Kpkm = 0.25;
	double kexpkm=0.0025;
	double klkpkm=0.012;
	double Vsd=0.03;

    int i, k, j, l; // counters

        k=1;
        do {  

pkmsn=((double)(k))/1000.0*runn;	

dv21dt = kexpkm*tltp*pkmd/Vsd-klkpkm*pkmsn + vtrbaspkm + kpkmon2*pkmsn*pkmsn/(pkmsn*pkmsn+Kpkm*Kpkm)-pkmsn/taupkm;


		value=100.0*dv21dt; // derivative * 100 scale factor


		   out1.println(pkmsn + "\t" + value);          	

            k++;
           } while (k < 1000);


      out1.close();

       }
}


import java.io.*;

public class winltp 

/* This program simulates the sloped LTP window in Figure 6 of the PKM manuscript. It simulates 40 heterogeneous synapses, differing only in a single rate constant. As a result, each synapse (spine compartment) has a different LTP window, and the sum of all these individual windows yields a sloped, peaked LTP window (red curve, Fig. 6). */

/* Most of the code is the same as in basicmod.java, which simulates the different protocols including STC and SCTC. Therefore, comments are mostly restricted to the code specific for generating the sloped window. */

 {  public static void main (String args[]) throws IOException {

    PrintWriter out2 = new PrintWriter(new FileWriter("wsend.txt"));

    double dt=0.0002; // high-res timestep (min)
    double delta=0.01; // low-res timestep (min)

    double recstart=1995.0; // Time to start writing data
    double stime=2000.0; // Time of stimulus onset

    double isi=5.0;      // Spacing between tetanic stimuli

//Time to end simulation and stop writing data
    double recend=2600.0; // FOR LTP STOP AND WRITE 5 H POST STIMULUS 

     int recintvl=20; // (delta) Intervals to record

    double tref; // Time since stimulus onset

/* Time offset for crosstagging expts. Defined as (time of strong at S2 for PRP's) – (time of weak at S1 for tag). So positive doff is weak before strong. tref2 is then tref – doff. */

/* For generating the window, doff is set to the most negative offset. Then a second variable, delt, is used to sweep through the different offsets, from negative through positive, thereby generating the window of the amount of LTP vs. the offset. */

     double doff=-200.0; 

	double delt;

	double tref2;

//PROGRAM VARIABLES

// CAMKII
	double ck2act;

// SYNAPTIC MAPK CASCADE
	double kfraf;
	double kfrafd;
	double f1up;
	double f1dn;
	double f2up;
	double f2dn;
	double f3up;
	double f3dn;
	double g1up;
	double g1dn;
	double g2up;
	double g2dn;
	double g3up;
	double g3dn;
	double g4up;
	double g4dn;
	double g5up;
	double g5dn;
	double g6up;
	double g6dn;

	double raf;
	double rafp;
	double mkk;
	double mkkp;
	double mkkpp;
	double erk;
	double erkp;
	double erkpp;
	double erkact;

// SYNAPTIC LTP TAG VARIABLES
	double tagp1;
	double tltp;

// SYNAPTIC LTD TAG VARIABLES
	double tagd1;
	double tagd2;
	double tltd;
	double ppact; 
	
// PRP, PKM variables
	double psit1;
	double psit2;
	double prp;

	double pkmd;
	double pkmsn;

// Dendritic variables, then synaptic weight variables

	double ckdact;
	double erkdact;
	double raf2;
	double rafp2;
	double mkk2;
	double mkkp2;
	double mkkpp2;
	double erk2;
	double erkp2;
	double erkpp2;

	double wsyn;
	double fsyn;
	double nsyn;

    	double dv1dt;
    	double dv2dt;
    	double dv3dt;
    	double dv4dt;
    	double dv5dt;
    	double dv6dt;
    	double dv7dt;
    	double dv8dt;
    	double dv9dt;
    	double dv10dt;
    	double dv11dt;
    	double dv12dt;
    	double dv13dt;
    	double dv14dt;
	double dv15dt;
	double dv16dt;
	double dv17dt;
	double dv18dt;
	double dv19dt;
	double dv20dt;
	double dv21dt;
	double dv22dt;
	double dv23dt;
	double dv24dt;
	double dv25dt;
	double dv26dt;

// Synaptic and dendritic Ca concentrations and powers
	double casyn;
	double cadend;
	double powca; // casyn, synapse 1
	double powcad; // cadend, Ca pool for PRP synthesis
	double powkc; // for camkii activation
	double powkc2; // for LTD phosphatase activation
	double powkcd; // Dendritic “camk” activation for PKM

// MODEL PARAMETERS FOLLOW:

	double Cabas=0.04;


	double AMPTETCA=1.4;
	double AMPCHEMCA=0.24;

	double AMPTETSTIM=0.006;
	double AMPCHEMSTIM=0.007; 

	double AMPSLCA=0.17;
	double AMPSLST=0.02;
	double AMPWLCA=0.16;
	double AMPWLST=0.02;

	double AMPTETCAD=0.65;
	double AMPCHEMCAD=0.24;

	double AMPTETSTIMD=0.03;
	double AMPCHEMSTIMD=0.007;

	double AMPSLCAD=0.17;
	double AMPSLSTD=0.017;
	double AMPWLCAD=0.04;
	double AMPWLSTD=0.006;

	double kfck2=200.0;
	double tauck2=1.0;
	double Kck2=1.4;

	double Kck2d=0.6;

	double kfpp=2.0;
	double taupp=2.0;
	double Kpp=0.225;

	double raftot=0.25;
	double mkktot=0.25;
	double erktot=0.25;

	double kbraf=0.12;
	double kfmkk=0.6;
	double kbmkk=0.025;
	double Kmkk=0.25;
	double kferk=0.52;
	double kberk=0.025;
	double Kmk=0.25;
	double kfbasraf=0.003;
	double kfbasrafd=0.003;
	double taufras=0.5;
	double tausras=4.0;

	double kphos1=0.45;
	double kdeph1=0.006;

	double kphos4=2.0;
	double kdeph4=0.011;
	double kphos5=0.011;
	double kdeph5=0.04;

	double kphos7=4.0;
	double kdeph7=0.1;
	double kphos8=0.015;
	double kdeph8=0.02;

	double ktrans1=2.2;
	double vtrbas1=0.001;
	double tauprp=45.0;

// PKM parameters
	double kpkmon=0.5;
	double kpkmon2=0.055;
	double taupkm=50.0;
	double vtrbaspkm=0.0003;
	double Kpkm = 0.75;
	double klkpkm=0.012;
	double Vsd=0.03;

/* The rate constant for movement of PKM into the spine, kexpkm, is the only parameter that varies between the 40 heterogeneous spines. It varies from its basal value, kexpkmbas. The equation for variation is given below, it uses sh1 and sh2 */
	double kexpkmbas=0.0025;
	double kexpkm;
	double sh1;
	double sh2;

	double kltp=0.014;
	double kltd=0.03;
	double tfsyn=30.0;
	double tnsyn=600.0;
	double vfbas=0.01;
	double vdbas=0.0033;

// Inhibition factors for enzymes and PRP's. 

	double inherk;
	double inhmkk;
	double inhck2;
	double inhprot;
	double inhpp;
	double inhpkm;

/* The following variable, wend, is used to store the synaptic weight at the ending time of each simulation. Commonly this is 200 minutes after the induction of LTP. This synaptic weight is outputted and used for the LTP windows in Fig. 6C. Note, the LTD window in Figure 6D is generated by a very similar program, except only 1 synapse, not 40, needs to be used so there is no loop over synapses (spines). */

	double wend;

// TIME STUFF, COUNTERS, VALUE ARRAY

    double time; // time (minutes)
    double timewrite;

    int i, k, j, l, mone, mtwo; // counters

    double[] values=new double[28];

// OUTER LOOP OVER 100 VALUES OF OFFSET BETWEEN S1 and S2 STIMULI

        mtwo=0;
        do {  

		time = 0.0;

		delt = doff+((double)(mtwo))*4.0; // with these numbers, the offset, delt, will range from -200 (i.e., the value of doff) to +200.

		wend=0.0;

// MIDDLE LOOP OVER 40 SYNAPSES (SPINES)

        mone=1;
        do {  

		time = 0.0;

/* This bit of code varies the rate constant kexpkm in regular steps over the 40 spines */
		sh1=(double)(mone);
		sh2=-30.0;
		kexpkm = kexpkmbas + kexpkmbas*1.0*(sh2+sh1)/40.0;

// VARIABLE INITIALIZATION. Use small but nonzero initial values
// to avoid extremely small numbers in output files.

	values[1]=0.001;
	values[2]=0.5*raftot;
	values[3]=0.3*mkktot;
	values[4]=0.4*mkktot;
	values[5]=0.3*erktot;
	values[6]=0.4*erktot;
	values[7]=0.001;
	values[8]=0.001;
	values[9]=0.001;
	values[10]=0.001;
	values[11]=0.001;
	values[12]=0.5*raftot;
	values[13]=0.3*mkktot;
	values[14]=0.4*mkktot;
	values[15]=0.3*erktot;
	values[16]=0.4*erktot;
	values[17]=0.001;
	values[18]=0.001;
	values[19]=vtrbas1*tauprp;
	values[20]=vtrbaspkm*taupkm;
	values[21]=0.001;
	values[22]=0.01;
	values[23]=0.01;
	values[24]=0.01;
	values[25]=0.01;

// MAIN SIMULATION LOOP (LARGER TIMESTEP)

        k=1;
        do {  

// INNER SIMULATION LOOP

            j=1;
            do {

		tref=time-stime;
		casyn=Cabas;
		cadend=Cabas;

		ck2act=values[1];

		raf=values[2];
		mkk=values[3];
		mkkpp=values[4];
		erk=values[5];
		erkpp=values[6];

		ppact=values[7];

		tagp1=values[8];

		tagd1=values[9];
		tagd2=values[10];

		ckdact=values[11];

		raf2=values[12];
		mkk2=values[13];
		mkkpp2=values[14];
		erk2=values[15];
		erkpp2=values[16];

		psit1=values[17];
		psit2=values[18];

		prp=values[19];

		pkmd=values[20];

		pkmsn=values[21];

		fsyn=values[22];

		nsyn=values[23];

		wsyn=values[24];

/* Synaptic variables are simulated at Synapse 1. */

       f1up=0.0;
       f1dn=1.0;
       f2up=0.0;
       f2dn=1.0;
       f3up=0.0;
       f3dn=1.0;
       g1up=0.0;
       g1dn=1.0;
       g2up=0.0;
       g2dn=1.0;
       g3up=0.0;
       g3dn=1.0;
       g4up=0.0;
       g4dn=1.0;
       g5up=0.0;
       g5dn=1.0;
       g6up=0.0;
       g6dn=1.0;
       kfraf=kfbasraf;
       kfrafd=kfbasrafd;

// Tetanic protocol
/*
		if (tref > 0.0 && tref < 0.05)
		  {
		  casyn=AMPTETCA;
		  cadend=AMPTETCAD;
		  }
		if (tref > (0.0+isi) && tref < (0.0+isi+0.05))
		  {
		  casyn=AMPTETCA;
		  cadend=AMPTETCAD;
		  }
		if (tref > (0.0+2.0*isi) && tref < (0.0+2.0*isi+0.05))
		  {
		  casyn=AMPTETCA;
		  cadend=AMPTETCAD;
		  }

		if (tref > 0.0 && tref < 60.0)
		  {
		  f1up = 1.0-Math.exp(-(tref)/taufras);
		  f1dn = (AMPTETSTIM-kfbasraf)* Math.exp(-(tref)/tausras);
		  }
		if (tref > (0.0+isi) && tref < 60.0)
		  {
		  f2up = 1.0-Math.exp(-(tref-isi)/taufras);
		  f2dn = (AMPTETSTIM-kfbasraf)* Math.exp(-(tref-isi)/tausras);
		  }
		if (tref > (0.0+2.0*isi) && tref < 60.0)
		  {
		  f3up = 1.0-Math.exp(-(tref-2.0*isi)/taufras);
		  f3dn = (AMPTETSTIM-kfbasraf)* Math.exp(-(tref-2.0*isi)/tausras);
		  }
		if (tref > 0.0 && tref < 60.0)
		  {
		  g1up = 1.0-Math.exp(-(tref)/taufras);
		  g1dn = (AMPTETSTIMD-kfbasrafd)* Math.exp(-(tref)/tausras);
		  }
		if (tref > (0.0+isi) && tref < 60.0)
		  {
		  g2up = 1.0-Math.exp(-(tref-isi)/taufras);
		  g2dn = (AMPTETSTIMD-kfbasrafd)* Math.exp(-(tref-isi)/tausras);
		  }
		if (tref > (0.0+2.0*isi) && tref < 60.0)
		  {
		  g3up = 1.0-Math.exp(-(tref-2.0*isi)/taufras);
		  g3dn = (AMPTETSTIMD-kfbasrafd)* Math.exp(-(tref-2.0*isi)/tausras);
		  }
*/
// Chem LTP
// Activates ERK
// Have to assume Chem treatment increases neuron excitability some and
// lets in some extra Ca, otherwise I dont get decent LLTP.
/*
		if (tref > 0.0 && tref < 30.0)
		  {
		  casyn=AMPCHEMCA;
		  cadend=AMPCHEMCAD;
		  }
		if (tref > 0.0 && tref < 60.0)
		  {
		  f1up= (AMPCHEMSTIM-kfbasraf)*(1.0-Math.exp(-(tref)/taufras));
		  if (tref > 30.0) {f1dn=Math.exp(-(tref-30.0)/tausras);}
		  }
		if (tref > 0.0 && tref < 60.0)
		  {
		  g1up= (AMPCHEMSTIMD-kfbasrafd)*(1.0-Math.exp(-(tref)/taufras));
		  if (tref > 30.0) {g1dn=Math.exp(-(tref-30.0)/tausras);}
		  }
*/

// WTET PROTOCOL

		if (tref > 0.0 && tref < 0.05)
		  {
		  casyn=AMPTETCA;
		  cadend=AMPTETCAD;
		  }
		if (tref > 0.0 && tref < 60.0)
		  {
		  f1up = 1.0-Math.exp(-(tref)/taufras);
		  f1dn = (AMPTETSTIM-kfbasraf)* Math.exp(-(tref)/tausras);
		  }
		if (tref > 0.0 && tref < 60.0)
		  {
		  g1up = 1.0-Math.exp(-(tref)/taufras);
		  g1dn = (AMPTETSTIMD-kfbasrafd)* Math.exp(-(tref)/tausras);
		  }


// SLFS STIMULUS TO PRODUCE L-LTD
/*
		if ((tref > 0.0) && (tref < 15.0))
		{
			 casyn=AMPSLCA;
			 cadend=AMPSLCAD;
		}
		if (tref > 0.0 && tref < 60.0)
		  {
		  f1up= (AMPSLST-kfbasraf)*(1.0-Math.exp(-(tref)/taufras));
		  if (tref > 15.0) {f1dn=Math.exp(-(tref-15.0)/tausras);}
		  }
		if (tref > 0.0 && tref < 60.0)
		  {
		  g1up= (AMPSLSTD-kfbasrafd)*(1.0-Math.exp(-(tref)/taufras));
		  if (tref > 15.0) {g1dn=Math.exp(-(tref-15.0)/tausras);}
		  }
*/
// WLFS STIMULUS
/*
		if ((tref > 0.0) && (tref < 15.0))
		{
			 casyn=AMPWLCA;
			 cadend=AMPWLCAD;
		}
		if (tref > 0.0 && tref < 60.0)
		  {
		  f1up= (AMPWLST-kfbasraf)*(1.0-Math.exp(-(tref)/taufras));
		  if (tref > 15.0) {f1dn=Math.exp(-(tref-15.0)/tausras);}
		  }
		if (tref > 0.0 && tref < 60.0)
		  {
		  g1up= (AMPWLSTD-kfbasrafd)*(1.0-Math.exp(-(tref)/taufras));
		  if (tref > 15.0) {g1dn=Math.exp(-(tref-15.0)/tausras);}
		  }
*/

/* NOW REPEAT these protocols for STC or SCTC simulations in which synapse 2 activates the dendrite. */

	tref2 = tref-delt; // Offsets PRP stimulus to be del minutes later than Tag stimulus at synapse 1.

// Tetanic protocol

		if (tref2 > 0.0 && tref2 < 0.05)
		  {
		  cadend=AMPTETCAD;
		  }
		if (tref2 > (0.0+isi) && tref2 < (0.0+isi+0.05))
		  {
		  cadend=AMPTETCAD;
		  }
		if (tref2 > (0.0+2.0*isi) && tref2 < (0.0+2.0*isi+0.05))
		  {
		  cadend=AMPTETCAD;
		  }
		if (tref2 > 0.0 && tref2 < 60.0)
		  {
		  g4up = 1.0-Math.exp(-(tref2)/taufras);
		  g4dn = (AMPTETSTIMD-kfbasrafd)* Math.exp(-(tref2)/tausras);
		  }
		if (tref2 > (0.0+isi) && tref2 < 60.0)
		  {
		  g5up = 1.0-Math.exp(-(tref2-isi)/taufras);
		  g5dn = (AMPTETSTIMD-kfbasrafd)* Math.exp(-(tref2-isi)/tausras);
		  }
		if (tref2 > (0.0+2.0*isi) && tref2 < 60.0)
		  {
		  g6up = 1.0-Math.exp(-(tref2-2.0*isi)/taufras);
		  g6dn = (AMPTETSTIMD-kfbasrafd)* Math.exp(-(tref2-2.0*isi)/tausras);
		  }


// SLFS STIMULUS TO PRODUCE L-LTD
/*
		if (tref2 > 0.0 && tref2 < 15.0 && cadend < AMPSLCAD)
		{
			 cadend=AMPSLCAD;
		}
		if (tref2 > 0.0 && tref2 < 60.0)
		  {
	 g4up= (AMPSLSTD-kfbasrafd)*(1.0-Math.exp(-(tref2)/taufras));
		  if (tref2 > 15.0) {g4dn=Math.exp(-(tref2-15.0)/tausras);}
		  }
*/

// GIVE COMPOSITE KFRAFS
		if (tref > -300.0 && tref < 300.0)
		  {
kfraf=kfbasraf + (f1up*f1dn+f2up*f2dn+f3up*f3dn);
kfrafd=kfbasrafd + (g1up*g1dn+g2up*g2dn+g3up*g3dn) + (g4up*g4dn+g5up*g5dn+g6up*g6dn);
		  }


// INHIBITOR APPLICATIONS. FIRST SET THE BASELINE NONINHIBITED FACTOR VALUES
// TO 1.

		inherk=1.0;
		inhck2=1.0;
		inhprot=1.0;
		inhmkk=1.0;
		inhpp=1.0;
		inhpkm=1.0;

		if (tref > 300.0 && tref < 360.0)
		  {

/* ERK inhibition, as of March 14, blocks both LLTD and LLTP, as it should. */
//			inherk=0.1; 

//			inhck2=0.1;

//			inhprot=0.25; 

//			inhmkk=0.1; 

//			inhpp=0.1; 

//			inhpkm=0.2;
		  }

// BEGINNING OF ACTUAL CODE FOR UPDATING DYNAMIC VARIABLES. 

// DEFINE SOME AUXILIARY FUNCTIONS
		powca=casyn*casyn*casyn*casyn;
		powcad=cadend*cadend*cadend*cadend;

		powkc=Kck2*Kck2*Kck2*Kck2;
		powkcd=Kck2d*Kck2d*Kck2d*Kck2d;
		powkc2=Kpp*Kpp*Kpp*Kpp;

		rafp=raftot-raf;
		mkkp=mkktot-mkk-mkkpp;
		erkp=erktot-erk-erkpp;

		rafp2=raftot-raf2;
		mkkp2=mkktot-mkk2-mkkpp2;
		erkp2=erktot-erk2-erkpp2;

		dv1dt = kfck2*(powca/(powca+powkc)) - ck2act/tauck2;

		dv2dt = -kfraf*raf+kbraf*rafp;

dv3dt = -inhmkk*kfmkk*rafp*mkk/(mkk+Kmkk)+kbmkk*mkkp/(mkkp+Kmkk); 

		dv4dt = inhmkk*kfmkk*rafp*mkkp/(mkkp+Kmkk)-kbmkk*mkkpp/(mkkpp+Kmkk);

		dv5dt = -kferk*mkkpp*erk/(erk+Kmk)
			+kberk*erkp/(erkp+Kmk);

		dv6dt = kferk*mkkpp*erkp/(erkp+Kmk)
			 -kberk*erkpp/(erkpp+Kmk);

		erkact = erkpp;

// ODE for LTD phosphatase activation

		dv7dt = kfpp*(powca/(powca+powkc2))-ppact/taupp;

// ODEs for tag phosphorylation, tag is at synapse 1

		dv8dt = kphos1*inhck2*ck2act*(1.0-tagp1) - kdeph1*tagp1;

/* Drive PRP synthesis by a different erk and camk activity. That is an easier way to simulate crosstagging at two synapses, without having to duplicate all the variables. I still have to duplicate everything down to ERK activation. */

// ODEs for Depression Tag Phospho Sites

		dv9dt = inherk*kphos4*erkact*(1.0-tagd1)-kdeph4*tagd1;

		dv10dt = inhpp*kdeph5*ppact*(1.0-tagd2)-kphos5*tagd2;

/* Synapse 1 is always considered as the tagged synapse, synapse 2 the prp synapse */

		tltp = tagp1*tagp1;
		tltd = tagd1*tagd2;

/* Important note – Feb 24 2011. I removed ERK from LTP TAG, from tltp. That is, the site tagp3 is not being used now! Because Sajikumar and Frey 2007 say ERK is NOT needed for setting the LTP tag. Instead it is needed for PRP synthesis. */

/* ODEs for dendrite. */

		dv11dt = kfck2*(powcad/(powcad+powkcd)) - ckdact/tauck2;

		dv12dt = -kfrafd*raf2+kbraf*rafp2;

		dv13dt = -inhmkk*kfmkk*rafp2*mkk2/(mkk2+Kmkk)+kbmkk*mkkp2/(mkkp2+Kmkk);

		dv14dt = inhmkk*kfmkk*rafp2*mkkp2/(mkkp2+Kmkk)-kbmkk*mkkpp2/(mkkpp2+Kmkk);

		dv15dt = -kferk*mkkpp2*erk2/(erk2+Kmk)
			+kberk*erkp2/(erkp2+Kmk);

		dv16dt = kferk*mkkpp2*erkp2/(erkp2+Kmk)
			 -kberk*erkpp2/(erkpp2+Kmk);

		erkdact = erkpp2;

// ODE for a dendritic ERK phosphor site.

		dv17dt = inherk*kphos7*erkdact*(1.0-psit1)-kdeph7*psit1;

// ODE for phosphorylation by camkii of site that governs PKM synthesis

		dv18dt = inhck2*kphos8*ckdact*(1.0-psit2)-kdeph8*psit2;

/* Following ODE is synthesis of a PRP that is needed for LTD */

		dv19dt = inhprot*ktrans1*psit1*psit1-prp/tauprp + inhprot*vtrbas1;

/* ODE for dendritic PKM */

		dv20dt = inhprot*kpkmon*psit1*psit2-pkmd/taupkm + inhprot*vtrbaspkm-kexpkm*tltp*pkmd+Vsd*klkpkm*pkmsn;

/* synaptic PKM */

dv21dt = kexpkm*tltp*pkmd/Vsd-klkpkm*pkmsn + inhprot*vtrbaspkm + kpkmon2*inhpkm*inhpkm*inhprot*pkmsn*pkmsn/(inhpkm*inhpkm*pkmsn*pkmsn+Kpkm*Kpkm)-pkmsn/taupkm;

// Fsyn ODE
		dv22dt = kltp*inhpkm*pkmsn-fsyn/tfsyn + vfbas; 

// nsyn ODE
		dv23dt = -kltd*tltd*prp*nsyn-nsyn/tnsyn + vdbas; 

		wsyn=fsyn*nsyn;
		values[24]=fsyn*nsyn;

		values[1]+=dt*dv1dt;
		values[2]+=dt*dv2dt;
		values[3]+=dt*dv3dt;
		values[4]+=dt*dv4dt;
		values[5]+=dt*dv5dt;
		values[6]+=dt*dv6dt;
		values[7]+=dt*dv7dt;
		values[8]+=dt*dv8dt;
		values[9]+=dt*dv9dt;
		values[10]+=dt*dv10dt;
		values[11]+=dt*dv11dt;
		values[12]+=dt*dv12dt;
		values[13]+=dt*dv13dt;
		values[14]+=dt*dv14dt;
		values[15]+=dt*dv15dt;
		values[16]+=dt*dv16dt;
		values[17]+=dt*dv17dt;
		values[18]+=dt*dv18dt;
		values[19]+=dt*dv19dt;
		values[20]+=dt*dv20dt;
		values[21]+=dt*dv21dt;
		values[22]+=dt*dv22dt;
		values[23]+=dt*dv23dt;

/* Equilibrate basal synaptic weight, prior to stimulation, according to basal kinase activities. */

		if ((tref < -1.0) && (tref2 < -1.0))
		  {
       fsyn = (kltp*pkmsn+vfbas)/(kltp*pkmsn+1.0/tfsyn);
       values[22] = (kltp*pkmsn+vfbas)/(kltp*pkmsn+1.0/tfsyn);
       
       nsyn = vdbas/(kltd*tltd*prp+1.0/tnsyn);
       values[23] = vdbas/(kltd*tltd*prp+1.0/tnsyn);

		  wsyn = fsyn*nsyn;
		  values[24] = fsyn*nsyn;
		  }


// Increment time
                time=time+dt;

// END INNER LOOP

                j++;
               } while (j <= delta/dt);

            k++;
           } while (k <= recend/delta);

/* Here, I am adding up all 40 of the individual spine weights and then dividing by 40 to obtain the composite weight, wend, corresponding to the “observed” synaptic weight as discussed in the PKM manuscript. */

		wend=wend+wsyn/40.0;

            mone++;
           } while (mone <= 40);

			out2.println(delt + "\t" + wend);

            mtwo++;
           } while (mtwo <= 100);

// END OF LOOPS AND OF SIMULATION. CLOSE OUTPUT FILES.

      out2.close();

       }
}


import java.io.*;

public class wsltp

/* This program generates the bistable, square LTP window in Fig. 6 of the PKM manuscript, for a single standard spine as the stimulus interval between WTET and STET, delt, is varied in an STC protocol. The program is identical to winltp.java, which generates the sloped LTP window in Fig. 6, except that in the current program there is only one spine, not 40 heterogeneous spines. See winltp.java for further comments. */

 {  public static void main (String args[]) throws IOException {

    PrintWriter out2 = new PrintWriter(new FileWriter("wsingltp.txt"));
    double dt=0.0002; // high-res timestep (min)
    double delta=0.01; // low-res timestep (min)

    double recstart=1995.0; // Time to start writing data
    double stime=2000.0; // Time of stimulus onset

    double isi=5.0;      // Spacing between tetanic stimuli

//Time to end simulation and stop writing data
    double recend=2600.0; // FOR LTP STOP AND WRITE 5 H POST STIMULUS

     int recintvl=20; // (delta) Intervals to record

    double tref; // Time since stimulus onset

/* Time offset for crosstagging expts. Defined as (time of strong at S2 for PRP's) – (time of weak at S1 for tag). So positive delt is weak before strong. tref2 is then tref – delt. */

     double doff=-200.0; 

	double delt;

	double tref2;

//PROGRAM VARIABLES

// CAMKII
	double ck2act;

// SYNAPTIC MAPK CASCADE
	double kfraf;
	double kfrafd;
	double f1up;
	double f1dn;
	double f2up;
	double f2dn;
	double f3up;
	double f3dn;
	double g1up;
	double g1dn;
	double g2up;
	double g2dn;
	double g3up;
	double g3dn;
	double g4up;
	double g4dn;
	double g5up;
	double g5dn;
	double g6up;
	double g6dn;

	double raf;
	double rafp;
	double mkk;
	double mkkp;
	double mkkpp;
	double erk;
	double erkp;
	double erkpp;
	double erkact;

// SYNAPTIC LTP TAG VARIABLES
	double tagp1;
	double tltp;

// SYNAPTIC LTD TAG VARIABLES
	double tagd1;
	double tagd2;
	double tltd;
	double ppact; // LTD phosphatase activity. Activate similar to CAMKII but with lower KD for casyn.
	
// PRP, PKM variables
	double psit1;
	double psit2;
	double prp;

	double pkmd;
	double pkmsn;

	double wsyn;
	double fsyn;
	double nsyn;

    	double dv1dt;
    	double dv2dt;
    	double dv3dt;
    	double dv4dt;
    	double dv5dt;
    	double dv6dt;
    	double dv7dt;
    	double dv8dt;
    	double dv9dt;
    	double dv10dt;
    	double dv11dt;
    	double dv12dt;
    	double dv13dt;
    	double dv14dt;
	double dv15dt;
	double dv16dt;
	double dv17dt;
	double dv18dt;
	double dv19dt;
	double dv20dt;
	double dv21dt;
	double dv22dt;
	double dv23dt;
	double dv24dt;
	double dv25dt;
	double dv26dt;

// Synaptic and dendritic Ca concentrations and powers
	double casyn;
	double cadend;
	double powca; // casyn, synapse 1
	double powcad; // cadend, Ca pool for PRP synthesis
	double powkc; // for camkii activation
	double powkc2; // for LTD phosphatase activation
	double powkcd; // Dendritic “camk” activation for PKM

// WORK IN CONCENTRATION UNITS OF uM, TIME UNITS OF MIN

// MODEL PARAMETERS FOLLOW:

/* BASAL CA, STIMULUS AMPLITUDES. These are for LTP protocols. */

	double Cabas=0.04;

/* FIRST GROUP OF AMPLITUDES ARE FOR TAG SETTING. THEY ARE AT SYNAPSE 1. SECOND GROUP OF AMPLITUDES, IS AT SYNAPSE 2 FOR A CROSSTAGGING EXPERIMENT, OR NEAR SYNAPSE 1 BUT AT SOME DISTANCE FROM THE PSD FOR A SINGLE SYNAPSE PROTOCOL. THAT SECOND GROUP OF LOWER AMPLITUDES DRIVES PRP SYNTHESIS. */

	double AMPTETCA=1.4;
	double AMPCHEMCA=0.24;

	double AMPTETSTIM=0.006;
	double AMPCHEMSTIM=0.007; 

/* March 16, 2011
HEY, LOOK, MY SYNAPTIC (not dendritic) erk activation amplitudes are same for all stimuli, all LTP inducing stimuli (except that CHEM is a little lower) and LFS LTD stimuli! Important! This means only the duration of ERK activation, not the amount, determines how much of the LTD tag is set! I can regard the amplitude of Ras stimulus (AMP parameters) as being the same regardless of stimulus! */

// LTD AMPLITUDES FOR CA AND RAS, DURING SLFS AND WLFS

	double AMPSLCA=0.17;
	double AMPSLST=0.02;
	double AMPWLCA=0.16;
	double AMPWLST=0.02;

/* SECOND GROUP OF AMPLITUDES, FOR CADEND AND KFRAFD POOLS. */

	double AMPTETCAD=0.65;
	double AMPCHEMCAD=0.24;

	double AMPTETSTIMD=0.03;
	double AMPCHEMSTIMD=0.007;

/* LTD AMPLITUDES group 2. BECAUSE SLFS VS. WLFS DIFFER ONLY IN THE STRENGTH, NOT THE LENGTH, OF THEIR STIMULUS, I REALLY HAVE TO HAVE THE CADEND AND KFRAFD LOWER FOR WLFS THAN FOR SLFS, SO THAT NO PRP'S ARE MADE AND ONLY THE TAG IS SET DURING WLFS */

	double AMPSLCAD=0.17;
	double AMPSLSTD=0.017;
	double AMPWLCAD=0.04;
	double AMPWLSTD=0.006;

// RATE CONSTANTS AND MICHAELIS CONSTANT FOR CAMKII 
	double kfck2=200.0;
	double tauck2=1.0;
	double Kck2=1.4;

/* LOWER BINDING CONSTANT FOR “DENDRITIC” CAMKII THAT DRIVES PKM SYN, SO THAT PKM CAN BE SYNTHESIZED BY SLFS */

	double Kck2d=0.6;

// RATE CONSTANTS AND Ca binding CONSTANT FOR PP
	double kfpp=2.0;
	double taupp=2.0;
	double Kpp=0.225;

//CONSERVED ENZYME AMOUNTS
	double raftot=0.25;
	double mkktot=0.25;
	double erktot=0.25;

// MAPK pathway rate and Michaelis constants
	double kbraf=0.12;
	double kfmkk=0.6;
	double kbmkk=0.025;
	double Kmkk=0.25;
	double kferk=0.52;
	double kberk=0.025;
	double Kmk=0.25;
	double kfbasraf=0.003;
	double kfbasrafd=0.003;
	double taufras=0.5;
	double tausras=4.0;

// Rate constants and other parameters
// describing LTP synaptic tagging.

	double kphos1=0.45;
	double kdeph1=0.006;

// Rate constants describing LTD synaptic tagging.

	double kphos4=2.0;
	double kdeph4=0.011;
	double kphos5=0.011;
	double kdeph5=0.04;

/* PHOS AND DEPHOS RATE PARAMS for sites that govern PRP synthesis.
Have to use these to time filter camk2 activity so its effect overlaps with erk activity effect. That is, if I use camk2 directly in prp ode, the duration of prp synthesis, like that of ck2 activation itself, is unreasonably short, and turns off before erk has chance to fully activate. */
	double kphos7=4.0;
	double kdeph7=0.1;
	double kphos8=0.015;
	double kdeph8=0.02;

// Maximal and basal transcription rates for PRP, decay of PRP
	double ktrans1=2.2;
	double vtrbas1=0.001;
	double tauprp=45.0;

// PKM parameters
	double kpkmon=0.5;
	double kpkmon2=0.055;
	double taupkm=50.0;
	double vtrbaspkm=0.0003;
	double Kpkm = 0.75;
	double kexpkm=0.0025;
	double klkpkm=0.012;
	double Vsd=0.03;

// Inhibition factors for enzymes and PRP's. 

	double inherk;
	double inhmkk;
	double inhck2;
	double inhprot;
	double inhpp;
	double inhpkm;

// Rate constants and other parameters for synaptic 
// weight changes, that is, changes in variable Wsyn 

	double kltp=0.014;
	double kltd=0.03;
	double tfsyn=30.0;
	double tnsyn=600.0;
	double vfbas=0.01;
	double vdbas=0.0033;

	double wend=0.0;

	double ckdact;
	double erkdact;
	double rafd;
	double rafp2;
	double mkk2;
	double mkkp2;
	double mkkpp2;
	double erk2;
	double erkp2;
	double erkpp2;

// TIME STUFF, COUNTERS, VALUE ARRAY

    double time; // time (minutes)
    double timewrite;

    int i, k, j, l, mone, mtwo; // counters

    double[] values=new double[28];

// OUTER LOOP OVER 100 VALUES OF OFFSET

        mtwo=0;
        do {  

		time = 0.0;

		delt = doff+((double)(mtwo))*4.0;

		time = 0.0;

// VARIABLE INITIALIZATION. Use small but nonzero initial values
// to avoid extremely small numbers in output files.

	values[1]=0.001;
	values[2]=0.5*raftot;
	values[3]=0.3*mkktot;
	values[4]=0.4*mkktot;
	values[5]=0.3*erktot;
	values[6]=0.4*erktot;
	values[7]=0.001;
	values[8]=0.001;
	values[9]=0.001;
	values[10]=0.001;
	values[11]=0.001;
	values[12]=0.5*raftot;
	values[13]=0.3*mkktot;
	values[14]=0.4*mkktot;
	values[15]=0.3*erktot;
	values[16]=0.4*erktot;
	values[17]=0.001;
	values[18]=0.001;
	values[19]=vtrbas1*tauprp;
	values[20]=vtrbaspkm*taupkm;
	values[21]=0.001;
	values[22]=0.01;
	values[23]=0.01;
	values[24]=0.01;
	values[25]=0.01;

// MAIN LOOP (LARGER TIMESTEP)

        k=1;
        do {  

// INNER SIMULATION LOOP

            j=1;
            do {

		tref=time-stime;
		casyn=Cabas;
		cadend=Cabas;

		ck2act=values[1];

		raf=values[2];
		mkk=values[3];
		mkkpp=values[4];
		erk=values[5];
		erkpp=values[6];

		ppact=values[7];

		tagp1=values[8];

		tagd1=values[9];
		tagd2=values[10];

		ckdact=values[11];

		rafd=values[12];
		mkk2=values[13];
		mkkpp2=values[14];
		erk2=values[15];
		erkpp2=values[16];

		psit1=values[17];
		psit2=values[18];

		prp=values[19];

		pkmd=values[20];

		pkmsn=values[21];

		fsyn=values[22];

		nsyn=values[23];

		wsyn=values[24];

/* Synaptic variables are simulated at Synapse 1. */


       f1up=0.0;
       f1dn=1.0;
       f2up=0.0;
       f2dn=1.0;
       f3up=0.0;
       f3dn=1.0;
       g1up=0.0;
       g1dn=1.0;
       g2up=0.0;
       g2dn=1.0;
       g3up=0.0;
       g3dn=1.0;
       g4up=0.0;
       g4dn=1.0;
       g5up=0.0;
       g5dn=1.0;
       g6up=0.0;
       g6dn=1.0;
       kfraf=kfbasraf;
       kfrafd=kfbasrafd;

// Tetanic protocol
/*
		if (tref > 0.0 && tref < 0.05)
		  {
		  casyn=AMPTETCA;
		  cadend=AMPTETCAD;
		  }
		if (tref > (0.0+isi) && tref < (0.0+isi+0.05))
		  {
		  casyn=AMPTETCA;
		  cadend=AMPTETCAD;
		  }
		if (tref > (0.0+2.0*isi) && tref < (0.0+2.0*isi+0.05))
		  {
		  casyn=AMPTETCA;
		  cadend=AMPTETCAD;
		  }

		if (tref > 0.0 && tref < 60.0)
		  {
		  f1up = 1.0-Math.exp(-(tref)/taufras);
		  f1dn = (AMPTETSTIM-kfbasraf)* Math.exp(-(tref)/tausras);
		  }
		if (tref > (0.0+isi) && tref < 60.0)
		  {
		  f2up = 1.0-Math.exp(-(tref-isi)/taufras);
		  f2dn = (AMPTETSTIM-kfbasraf)* Math.exp(-(tref-isi)/tausras);
		  }
		if (tref > (0.0+2.0*isi) && tref < 60.0)
		  {
		  f3up = 1.0-Math.exp(-(tref-2.0*isi)/taufras);
		  f3dn = (AMPTETSTIM-kfbasraf)* Math.exp(-(tref-2.0*isi)/tausras);
		  }
		if (tref > 0.0 && tref < 60.0)
		  {
		  g1up = 1.0-Math.exp(-(tref)/taufras);
		  g1dn = (AMPTETSTIMD-kfbasrafd)* Math.exp(-(tref)/tausras);
		  }
		if (tref > (0.0+isi) && tref < 60.0)
		  {
		  g2up = 1.0-Math.exp(-(tref-isi)/taufras);
		  g2dn = (AMPTETSTIMD-kfbasrafd)* Math.exp(-(tref-isi)/tausras);
		  }
		if (tref > (0.0+2.0*isi) && tref < 60.0)
		  {
		  g3up = 1.0-Math.exp(-(tref-2.0*isi)/taufras);
		  g3dn = (AMPTETSTIMD-kfbasrafd)* Math.exp(-(tref-2.0*isi)/tausras);
		  }
*/
// Chem LTP
// Activates ERK
// Have to assume Chem treatment increases neuron excitability some and
// lets in some extra Ca, otherwise I dont get decent LLTP.
/*
		if (tref > 0.0 && tref < 30.0)
		  {
		  casyn=AMPCHEMCA;
		  cadend=AMPCHEMCAD;
		  }
		if (tref > 0.0 && tref < 60.0)
		  {
		  f1up= (AMPCHEMSTIM-kfbasraf)*(1.0-Math.exp(-(tref)/taufras));
		  if (tref > 30.0) {f1dn=Math.exp(-(tref-30.0)/tausras);}
		  }
		if (tref > 0.0 && tref < 60.0)
		  {
		  g1up= (AMPCHEMSTIMD-kfbasrafd)*(1.0-Math.exp(-(tref)/taufras));
		  if (tref > 30.0) {g1dn=Math.exp(-(tref-30.0)/tausras);}
		  }
*/

// WTET PROTOCOL

		if (tref > 0.0 && tref < 0.05)
		  {
		  casyn=AMPTETCA;
		  cadend=AMPTETCAD;
		  }
		if (tref > 0.0 && tref < 60.0)
		  {
		  f1up = 1.0-Math.exp(-(tref)/taufras);
		  f1dn = (AMPTETSTIM-kfbasraf)* Math.exp(-(tref)/tausras);
		  }
		if (tref > 0.0 && tref < 60.0)
		  {
		  g1up = 1.0-Math.exp(-(tref)/taufras);
		  g1dn = (AMPTETSTIMD-kfbasrafd)* Math.exp(-(tref)/tausras);
		  }


// SLFS STIMULUS TO PRODUCE L-LTD
/*
		if ((tref > 0.0) && (tref < 15.0))
		{
			 casyn=AMPSLCA;
			 cadend=AMPSLCAD;
		}
		if (tref > 0.0 && tref < 60.0)
		  {
		  f1up= (AMPSLST-kfbasraf)*(1.0-Math.exp(-(tref)/taufras));
		  if (tref > 15.0) {f1dn=Math.exp(-(tref-15.0)/tausras);}
		  }
		if (tref > 0.0 && tref < 60.0)
		  {
		  g1up= (AMPSLSTD-kfbasrafd)*(1.0-Math.exp(-(tref)/taufras));
		  if (tref > 15.0) {g1dn=Math.exp(-(tref-15.0)/tausras);}
		  }
*/
// WLFS STIMULUS
/*
		if ((tref > 0.0) && (tref < 15.0))
		{
			 casyn=AMPWLCA;
			 cadend=AMPWLCAD;
		}
		if (tref > 0.0 && tref < 60.0)
		  {
		  f1up= (AMPWLST-kfbasraf)*(1.0-Math.exp(-(tref)/taufras));
		  if (tref > 15.0) {f1dn=Math.exp(-(tref-15.0)/tausras);}
		  }
		if (tref > 0.0 && tref < 60.0)
		  {
		  g1up= (AMPWLSTD-kfbasrafd)*(1.0-Math.exp(-(tref)/taufras));
		  if (tref > 15.0) {g1dn=Math.exp(-(tref-15.0)/tausras);}
		  }
*/

/* NOW REPEAT these protocols for STC or SCTC simulations in which synapse 2 activates the dendrite. */

	tref2 = tref-delt; // Offsets PRP stimulus to be del minutes later than Tag stimulus at synapse 1.

// Tetanic protocol

		if (tref2 > 0.0 && tref2 < 0.05)
		  {
		  cadend=AMPTETCAD;
		  }
		if (tref2 > (0.0+isi) && tref2 < (0.0+isi+0.05))
		  {
		  cadend=AMPTETCAD;
		  }
		if (tref2 > (0.0+2.0*isi) && tref2 < (0.0+2.0*isi+0.05))
		  {
		  cadend=AMPTETCAD;
		  }
		if (tref2 > 0.0 && tref2 < 60.0)
		  {
		  g4up = 1.0-Math.exp(-(tref2)/taufras);
		  g4dn = (AMPTETSTIMD-kfbasrafd)* Math.exp(-(tref2)/tausras);
		  }
		if (tref2 > (0.0+isi) && tref2 < 60.0)
		  {
		  g5up = 1.0-Math.exp(-(tref2-isi)/taufras);
		  g5dn = (AMPTETSTIMD-kfbasrafd)* Math.exp(-(tref2-isi)/tausras);
		  }
		if (tref2 > (0.0+2.0*isi) && tref2 < 60.0)
		  {
		  g6up = 1.0-Math.exp(-(tref2-2.0*isi)/taufras);
		  g6dn = (AMPTETSTIMD-kfbasrafd)* Math.exp(-(tref2-2.0*isi)/tausras);
		  }


// SLFS STIMULUS TO PRODUCE L-LTD
/*
		if (tref2 > 0.0 && tref2 < 15.0 && cadend < AMPSLCAD)
		{
			 cadend=AMPSLCAD;
		}
		if (tref2 > 0.0 && tref2 < 60.0)
		  {
	 g4up= (AMPSLSTD-kfbasrafd)*(1.0-Math.exp(-(tref2)/taufras));
		  if (tref2 > 15.0) {g4dn=Math.exp(-(tref2-15.0)/tausras);}
		  }
*/

// GIVE COMPOSITE KFRAFS
		if (tref > -300.0 && tref < 300.0)
		  {
kfraf=kfbasraf + (f1up*f1dn+f2up*f2dn+f3up*f3dn);
kfrafd=kfbasrafd + (g1up*g1dn+g2up*g2dn+g3up*g3dn) + (g4up*g4dn+g5up*g5dn+g6up*g6dn);
		  }


// INHIBITOR APPLICATIONS. FIRST SET THE BASELINE NONINHIBITED FACTOR VALUES
// TO 1.

		inherk=1.0;
		inhck2=1.0;
		inhprot=1.0;
		inhmkk=1.0;
		inhpp=1.0;
		inhpkm=1.0;

		if (tref > 300.0 && tref < 360.0)
		  {

/* ERK inhibition, as of March 14, blocks both LLTD and LLTP, as it should. */
//			inherk=0.1; 

//			inhck2=0.1;

//			inhprot=0.25; 

//			inhmkk=0.1; 

//			inhpp=0.1; 

//			inhpkm=0.1;
		  }

// BEGINNING OF ACTUAL CODE FOR UPDATING DYNAMIC VARIABLES. 

// DEFINE SOME AUXILIARY FUNCTIONS
		powca=casyn*casyn*casyn*casyn;
		powcad=cadend*cadend*cadend*cadend;

		powkc=Kck2*Kck2*Kck2*Kck2;
		powkcd=Kck2d*Kck2d*Kck2d*Kck2d;
		powkc2=Kpp*Kpp*Kpp*Kpp;

		rafp=raftot-raf;
		mkkp=mkktot-mkk-mkkpp;
		erkp=erktot-erk-erkpp;

		rafp2=raftot-rafd;
		mkkp2=mkktot-mkk2-mkkpp2;
		erkp2=erktot-erk2-erkpp2;

// Activation of CAMKII by a power of synaptic calcium, hill function,
// uses fourth powers.

		dv1dt = kfck2*(powca/(powca+powkc)) - ck2act/tauck2;

		dv2dt = -kfraf*raf+kbraf*rafp;

	dv3dt = -inhmkk*kfmkk*rafp*mkk/(mkk+Kmkk)+kbmkk*mkkp/(mkkp+Kmkk); 

		dv4dt = inhmkk*kfmkk*rafp*mkkp/(mkkp+Kmkk)-kbmkk*mkkpp/(mkkpp+Kmkk);

		dv5dt = -kferk*mkkpp*erk/(erk+Kmk)
			+kberk*erkp/(erkp+Kmk);

		dv6dt = kferk*mkkpp*erkp/(erkp+Kmk)
			 -kberk*erkpp/(erkpp+Kmk);

		erkact = erkpp;

// ODE for LTD phosphatase activation

		dv7dt = kfpp*(powca/(powca+powkc2))-ppact/taupp;

// ODEs for tag phosphorylation, tag is at synapse 1

		dv8dt = kphos1*inhck2*ck2act*(1.0-tagp1) - kdeph1*tagp1;

/* Drive PRP synthesis by a different erk and camk activity. That is an easier way to simulate crosstagging at two synapses, without having to duplicate all the variables. I still have to duplicate everything down to ERK activation. */

// ODEs for Depression Tag Phospho Sites

		dv9dt = inherk*kphos4*erkact*(1.0-tagd1)-kdeph4*tagd1;

		dv10dt = inhpp*kdeph5*ppact*(1.0-tagd2)-kphos5*tagd2;

/* Synapse 1 is always considered as the tagged synapse, synapse 2 the prp synapse */

		tltp = tagp1*tagp1;
		tltd = tagd1*tagd2;

/* Important note – Feb 24 2011. I removed ERK from LTP TAG, from tltp. That is, the site tagp3 is not being used now! Because Sajikumar and Frey 2007 say ERK is NOT needed for setting the LTP tag. Instead it is needed for PRP synthesis. */

/* ODEs for dendrite. */

		dv11dt = kfck2*(powcad/(powcad+powkcd)) - ckdact/tauck2;

		dv12dt = -kfrafd*rafd+kbraf*rafp2;

dv13dt = -inhmkk*kfmkk*rafp2*mkk2/(mkk2+Kmkk)+kbmkk*mkkp2/(mkkp2+Kmkk);

		dv14dt = inhmkk*kfmkk*rafp2*mkkp2/(mkkp2+Kmkk)-kbmkk*mkkpp2/(mkkpp2+Kmkk);

		dv15dt = -kferk*mkkpp2*erk2/(erk2+Kmk)
			+kberk*erkp2/(erkp2+Kmk);

		dv16dt = kferk*mkkpp2*erkp2/(erkp2+Kmk)
			 -kberk*erkpp2/(erkpp2+Kmk);

		erkdact = erkpp2;

// ODE for a dendritic ERK phosphor site.

		dv17dt = inherk*kphos7*erkdact*(1.0-psit1)-kdeph7*psit1;

// ODE for phosphorylation by camkii of site that governs PKM synthesis

		dv18dt = inhck2*kphos8*ckdact*(1.0-psit2)-kdeph8*psit2;

/* Following ODE is synthesis of a PRP that is needed for LTD */

		dv19dt = inhprot*ktrans1*psit1*psit1-prp/tauprp + inhprot*vtrbas1;

/* ODE for dendritic PKM */

		dv20dt = inhprot*kpkmon*psit1*psit2-pkmd/taupkm + inhprot*vtrbaspkm-kexpkm*tltp*pkmd+Vsd*klkpkm*pkmsn;

/* synaptic PKM */

dv21dt = kexpkm*tltp*pkmd/Vsd-klkpkm*pkmsn + inhprot*vtrbaspkm + kpkmon2*inhpkm*inhpkm*inhprot*pkmsn*pkmsn/(inhpkm*inhpkm*pkmsn*pkmsn+Kpkm*Kpkm)-pkmsn/taupkm;

// Fsyn ODE
		dv22dt = kltp*inhpkm*pkmsn-fsyn/tfsyn + vfbas; 

// nsyn ODE
		dv23dt = -kltd*tltd*prp*nsyn-nsyn/tnsyn + vdbas; 

		wsyn=fsyn*nsyn;
		values[24]=fsyn*nsyn;

		values[1]+=dt*dv1dt;
		values[2]+=dt*dv2dt;
		values[3]+=dt*dv3dt;
		values[4]+=dt*dv4dt;
		values[5]+=dt*dv5dt;
		values[6]+=dt*dv6dt;
		values[7]+=dt*dv7dt;
		values[8]+=dt*dv8dt;
		values[9]+=dt*dv9dt;
		values[10]+=dt*dv10dt;
		values[11]+=dt*dv11dt;
		values[12]+=dt*dv12dt;
		values[13]+=dt*dv13dt;
		values[14]+=dt*dv14dt;
		values[15]+=dt*dv15dt;
		values[16]+=dt*dv16dt;
		values[17]+=dt*dv17dt;
		values[18]+=dt*dv18dt;
		values[19]+=dt*dv19dt;
		values[20]+=dt*dv20dt;
		values[21]+=dt*dv21dt;
		values[22]+=dt*dv22dt;
		values[23]+=dt*dv23dt;

/* Equilibrate basal synaptic weight, prior to stimulation, according to basal kinase activities. */

		if ((tref < -1.0) && (tref2 < -1.0))
		  {
       fsyn = (kltp*pkmsn+vfbas)/(kltp*pkmsn+1.0/tfsyn);
       values[22] = (kltp*pkmsn+vfbas)/(kltp*pkmsn+1.0/tfsyn);
       
       nsyn = vdbas/(kltd*tltd*prp+1.0/tnsyn);
       values[23] = vdbas/(kltd*tltd*prp+1.0/tnsyn);

		  wsyn = fsyn*nsyn;
		  values[24] = fsyn*nsyn;
		  }

// Increment time
                time=time+dt;

// END INNER LOOP

                j++;
               } while (j <= delta/dt);

            k++;
           } while (k <= recend/delta);

		wend=wsyn;

			out2.println(delt + "\t" + wend);

            mtwo++;
           } while (mtwo <= 100);

// END OF LOOPS AND OF SIMULATION. CLOSE OUTPUT FILES.

      out2.close();

       }
}


import java.io.*;

public class windep 

/* This program generates the LTD window in Figure 6 of the PKM manuscript. It is similar to winltp.java, the program for the LTP window, with two changes, 1) only a single spine is simulated, so no loop over 40 spines, and 2) the stimulus with comments removed is SLFS, to induce LTD, rather than STET, to induce LTP. Since there is no other difference, see winltp.java and basicmod.java for further comments. */

 {  public static void main (String args[]) throws IOException {

    PrintWriter out2 = new PrintWriter(new FileWriter("wsendltd.txt"));

    double dt=0.0002; // high-res timestep (min)
    double delta=0.01; // low-res timestep (min)

    double recstart=1995.0; // Time to start writing data
    double stime=2000.0; // Time of stimulus onset

    double isi=5.0;      // Spacing between tetanic stimuli

//Time to end simulation and stop writing data

    double recend; 
/* FOR LTD WINDOW, RECEND IS SET LATER. IT IS 180 MINUTES AFTER THE END OF EITHER WLFS OR SLFS, WHICHEVER COMES SECOND. THAT'S WHEN THE DEGREE OF LTD IS WRITTEN OUT */

     int recintvl=20; // (delta) Intervals to record

    double tref; // Time since stimulus onset

/* Time offset for crosstagging expts. Defined as (time of strong at S2 for PRP's) – (time of weak at S1 for tag). So positive delt is weak before strong. tref2 is then tref – delt. */

     double doff=-222.0; 

	double delt;

	double tref2;

//PROGRAM VARIABLES

// CAMKII
	double ck2act;

// SYNAPTIC MAPK CASCADE
	double kfraf;
	double kfrafd;
	double f1up;
	double f1dn;
	double f2up;
	double f2dn;
	double f3up;
	double f3dn;
	double g1up;
	double g1dn;
	double g2up;
	double g2dn;
	double g3up;
	double g3dn;
	double g4up;
	double g4dn;
	double g5up;
	double g5dn;
	double g6up;
	double g6dn;

	double raf;
	double rafp;
	double mkk;
	double mkkp;
	double mkkpp;
	double erk;
	double erkp;
	double erkpp;
	double erkact;


// SYNAPTIC LTP TAG VARIABLES
	double tagp1;
	double tltp;

// SYNAPTIC LTD TAG VARIABLES
	double tagd1;
	double tagd2;
	double tltd;
	double ppact; // LTD phosphatase activity. Activate similar to CAMKII but with lower KD for casyn.
	
// PRP, PKM variables
	double psit1;
	double psit2;
	double prp;

	double pkmd;
	double pkmsn;

	double wsyn;
	double fsyn;
	double nsyn;

    	double dv1dt;
    	double dv2dt;
    	double dv3dt;
    	double dv4dt;
    	double dv5dt;
    	double dv6dt;
    	double dv7dt;
    	double dv8dt;
    	double dv9dt;
    	double dv10dt;
    	double dv11dt;
    	double dv12dt;
    	double dv13dt;
    	double dv14dt;
	double dv15dt;
	double dv16dt;
	double dv17dt;
	double dv18dt;
	double dv19dt;
	double dv20dt;
	double dv21dt;
	double dv22dt;
	double dv23dt;
	double dv24dt;
	double dv25dt;
	double dv26dt;

// Synaptic and dendritic Ca concentrations and powers
	double casyn;
	double cadend;
	double powca; // casyn, synapse 1
	double powcad; // cadend, Ca pool for PRP synthesis
	double powkc; // for camkii activation
	double powkc2; // for LTD phosphatase activation
	double powkcd; // Dendritic “camk” activation for PKM

// WORK IN CONCENTRATION UNITS OF uM, TIME UNITS OF MIN

// MODEL PARAMETERS FOLLOW:

/* BASAL CA, STIMULUS AMPLITUDES. These are for LTP protocols. */

	double Cabas=0.04;

/* FIRST GROUP OF AMPLITUDES ARE FOR TAG SETTING. THEY ARE AT SYNAPSE 1. SECOND GROUP OF AMPLITUDES, IS AT SYNAPSE 2 FOR A CROSSTAGGING EXPERIMENT, OR NEAR SYNAPSE 1 BUT AT SOME DISTANCE FROM THE PSD FOR A SINGLE SYNAPSE PROTOCOL. THAT SECOND GROUP OF LOWER AMPLITUDES DRIVES PRP SYNTHESIS. */

	double AMPTETCA=1.4;
	double AMPCHEMCA=0.24;

	double AMPTETSTIM=0.006;
	double AMPCHEMSTIM=0.007; 

/* March 16, 2011
HEY, LOOK, MY SYNAPTIC (not dendritic) erk activation amplitudes are same for all stimuli, all LTP inducing stimuli (except that CHEM is a little lower) and LFS LTD stimuli! Important! This means only the duration of ERK activation, not the amount, determines how much of the LTD tag is set! I can regard the amplitude of Ras stimulus (AMP parameters) as being the same regardless of stimulus! */

// LTD AMPLITUDES FOR CA AND RAS, DURING SLFS AND WLFS

	double AMPSLCA=0.17;
	double AMPSLST=0.02;
	double AMPWLCA=0.16;
	double AMPWLST=0.02;

/* SECOND GROUP OF AMPLITUDES, FOR CADEND AND KFRAFD POOLS. */

	double AMPTETCAD=0.65;
	double AMPCHEMCAD=0.24;

	double AMPTETSTIMD=0.03;
	double AMPCHEMSTIMD=0.007;

/* LTD AMPLITUDES group 2. BECAUSE SLFS VS. WLFS DIFFER ONLY IN THE STRENGTH, NOT THE LENGTH, OF THEIR STIMULUS, I REALLY HAVE TO HAVE THE CADEND AND KFRAFD LOWER FOR WLFS THAN FOR SLFS, SO THAT NO PRP'S ARE MADE AND ONLY THE TAG IS SET DURING WLFS */

	double AMPSLCAD=0.17;
	double AMPSLSTD=0.017;
	double AMPWLCAD=0.04;
	double AMPWLSTD=0.006;

// RATE CONSTANTS AND MICHAELIS CONSTANT FOR CAMKII 
	double kfck2=200.0;
	double tauck2=1.0;
	double Kck2=1.4;

/* LOWER BINDING CONSTANT FOR “DENDRITIC” CAMKII THAT DRIVES PKM SYN, SO THAT PKM CAN BE SYNTHESIZED BY SLFS */

	double Kck2d=0.6;

// RATE CONSTANTS AND Ca binding CONSTANT FOR PP
	double kfpp=2.0;
	double taupp=2.0;
	double Kpp=0.225;

//CONSERVED ENZYME AMOUNTS
	double raftot=0.25;
	double mkktot=0.25;
	double erktot=0.25;

// MAPK pathway rate and Michaelis constants
	double kbraf=0.12;
	double kfmkk=0.6;
	double kbmkk=0.025;
	double Kmkk=0.25;
	double kferk=0.52;
	double kberk=0.025;
	double Kmk=0.25;
	double kfbasraf=0.003;
	double kfbasrafd=0.003;
	double taufras=0.5;
	double tausras=4.0;
	double erksmult=1.0; /* These factors, and pkmmult below, are set to 1 and don't actually do anything */
	double erkdmult=1.0;

// Rate constants and other parameters
// describing LTP synaptic tagging.

	double kphos1=0.45;
	double kdeph1=0.006;

// Rate constants describing LTD synaptic tagging.

	double kphos4=2.0;
	double kdeph4=0.011;
	double kphos5=0.011;
	double kdeph5=0.04;

/* PHOS AND DEPHOS RATE PARAMS for sites that govern PRP synthesis.
Have to use these to time filter camk2 activity so its effect overlaps with erk activity effect. That is, if I use camk2 directly in prp ode, the duration of prp synthesis, like that of ck2 activation itself, is unreasonably short, and turns off before erk has chance to fully activate. */
	double kphos7=4.0;
	double kdeph7=0.1;
	double kphos8=0.015;
	double kdeph8=0.02;

// Maximal and basal transcription rates for PRP, decay of PRP
	double ktrans1=2.2;
	double vtrbas1=0.001;
	double tauprp=45.0;

// PKM parameters
	double kpkmon=0.5;
	double kpkmon2=0.055;
	double taupkm=50.0;
	double vtrbaspkm=0.0003;
	double Kpkm = 0.75;
	double kexpkm=0.0025;
	double klkpkm=0.012;
	double Vsd=0.03;

	double pkmmult=1.0;

// Inhibition factors for enzymes and PRP's. 

	double inherk;
	double inhmkk;
	double inhck2;
	double inhprot;
	double inhpp;
	double inhpkm;

// Rate constants and other parameters for synaptic 
// weight changes, that is, changes in variable Wsyn 

	double kltp=0.014;
	double kltd=0.03;
	double tfsyn=30.0;
	double tnsyn=600.0;
	double vfbas=0.01;
	double vdbas=0.0033;

	double wend=0.0;

	double ckdact;
	double erkdact;
	double raf2;
	double rafp2;
	double mkk2;
	double mkkp2;
	double mkkpp2;
	double erk2;
	double erkp2;
	double erkpp2;

// TIME STUFF, COUNTERS, VALUE ARRAY

    double time; // time (minutes)
    double timewrite;

    int i, k, j, l, mone, mtwo; // counters

    double[] values=new double[28];

// OUTER LOOP OVER 120 VALUES OF OFFSET

        mtwo=0;
        do {  

		time = 0.0;

		delt = doff+((double)(mtwo))*4.0;
		recend = stime+195.0;
		if (delt < 0.0) {recend = stime-delt+195.0;}


// VARIABLE INITIALIZATION. Use small but nonzero initial values
// to avoid extremely small numbers in output files.

	values[1]=0.001;
	values[2]=0.5*raftot;
	values[3]=0.3*mkktot;
	values[4]=0.4*mkktot;
	values[5]=0.3*erktot;
	values[6]=0.4*erktot;
	values[7]=0.001;
	values[8]=0.001;
	values[9]=0.001;
	values[10]=0.001;
	values[11]=0.001;
	values[12]=0.5*raftot;
	values[13]=0.3*mkktot;
	values[14]=0.4*mkktot;
	values[15]=0.3*erktot;
	values[16]=0.4*erktot;
	values[17]=0.001;
	values[18]=0.001;
	values[19]=vtrbas1*tauprp;
	values[20]=vtrbaspkm*taupkm;
	values[21]=0.001;
	values[22]=0.01;
	values[23]=0.01;
	values[24]=0.01;
	values[25]=0.01;

// MAIN LOOP (LARGER TIMESTEP)

        k=1;
        do {  

// INNER SIMULATION LOOP

            j=1;
            do {

		tref=time-stime;
		casyn=Cabas;
		cadend=Cabas;

		ck2act=values[1];

		raf=values[2];
		mkk=values[3];
		mkkpp=values[4];
		erk=values[5];
		erkpp=values[6];

		ppact=values[7];

		tagp1=values[8];

		tagd1=values[9];
		tagd2=values[10];

		ckdact=values[11];

		raf2=values[12];
		mkk2=values[13];
		mkkpp2=values[14];
		erk2=values[15];
		erkpp2=values[16];

		psit1=values[17];
		psit2=values[18];

		prp=values[19];

		pkmd=values[20];

		pkmsn=values[21];

		fsyn=values[22];

		nsyn=values[23];

		wsyn=values[24];

/* Synaptic variables are simulated at Synapse 1. */


       f1up=0.0;
       f1dn=1.0;
       f2up=0.0;
       f2dn=1.0;
       f3up=0.0;
       f3dn=1.0;
       g1up=0.0;
       g1dn=1.0;
       g2up=0.0;
       g2dn=1.0;
       g3up=0.0;
       g3dn=1.0;
       g4up=0.0;
       g4dn=1.0;
       g5up=0.0;
       g5dn=1.0;
       g6up=0.0;
       g6dn=1.0;
       kfraf=kfbasraf;
       kfrafd=kfbasrafd;

// Tetanic protocol
/*
		if (tref > 0.0 && tref < 0.05)
		  {
		  casyn=AMPTETCA;
		  cadend=AMPTETCAD;
		  }
		if (tref > (0.0+isi) && tref < (0.0+isi+0.05))
		  {
		  casyn=AMPTETCA;
		  cadend=AMPTETCAD;
		  }
		if (tref > (0.0+2.0*isi) && tref < (0.0+2.0*isi+0.05))
		  {
		  casyn=AMPTETCA;
		  cadend=AMPTETCAD;
		  }

		if (tref > 0.0 && tref < 60.0)
		  {
		  f1up = 1.0-Math.exp(-(tref)/taufras);
		  f1dn = (AMPTETSTIM-kfbasraf)* Math.exp(-(tref)/tausras);
		  }
		if (tref > (0.0+isi) && tref < 60.0)
		  {
		  f2up = 1.0-Math.exp(-(tref-isi)/taufras);
		  f2dn = (AMPTETSTIM-kfbasraf)* Math.exp(-(tref-isi)/tausras);
		  }
		if (tref > (0.0+2.0*isi) && tref < 60.0)
		  {
		  f3up = 1.0-Math.exp(-(tref-2.0*isi)/taufras);
		  f3dn = (AMPTETSTIM-kfbasraf)* Math.exp(-(tref-2.0*isi)/tausras);
		  }
		if (tref > 0.0 && tref < 60.0)
		  {
		  g1up = 1.0-Math.exp(-(tref)/taufras);
		  g1dn = (AMPTETSTIMD-kfbasrafd)* Math.exp(-(tref)/tausras);
		  }
		if (tref > (0.0+isi) && tref < 60.0)
		  {
		  g2up = 1.0-Math.exp(-(tref-isi)/taufras);
		  g2dn = (AMPTETSTIMD-kfbasrafd)* Math.exp(-(tref-isi)/tausras);
		  }
		if (tref > (0.0+2.0*isi) && tref < 60.0)
		  {
		  g3up = 1.0-Math.exp(-(tref-2.0*isi)/taufras);
		  g3dn = (AMPTETSTIMD-kfbasrafd)* Math.exp(-(tref-2.0*isi)/tausras);
		  }
*/
// Chem LTP
// Activates ERK
// Have to assume Chem treatment increases neuron excitability some and
// lets in some extra Ca, otherwise I dont get decent LLTP.
/*
		if (tref > 0.0 && tref < 30.0)
		  {
		  casyn=AMPCHEMCA;
		  cadend=AMPCHEMCAD;
		  }
		if (tref > 0.0 && tref < 60.0)
		  {
		  f1up= (AMPCHEMSTIM-kfbasraf)*(1.0-Math.exp(-(tref)/taufras));
		  if (tref > 30.0) {f1dn=Math.exp(-(tref-30.0)/tausras);}
		  }
		if (tref > 0.0 && tref < 60.0)
		  {
		  g1up= (AMPCHEMSTIMD-kfbasrafd)*(1.0-Math.exp(-(tref)/taufras));
		  if (tref > 30.0) {g1dn=Math.exp(-(tref-30.0)/tausras);}
		  }
*/

// WTET PROTOCOL
/*
		if (tref > 0.0 && tref < 0.05)
		  {
		  casyn=AMPTETCA;
		  cadend=AMPTETCAD;
		  }
		if (tref > 0.0 && tref < 60.0)
		  {
		  f1up = 1.0-Math.exp(-(tref)/taufras);
		  f1dn = (AMPTETSTIM-kfbasraf)* Math.exp(-(tref)/tausras);
		  }
		if (tref > 0.0 && tref < 60.0)
		  {
		  g1up = 1.0-Math.exp(-(tref)/taufras);
		  g1dn = (AMPTETSTIMD-kfbasrafd)* Math.exp(-(tref)/tausras);
		  }
*/

// SLFS STIMULUS TO PRODUCE L-LTD
/*
		if ((tref > 0.0) && (tref < 15.0))
		{
			 casyn=AMPSLCA;
			 cadend=AMPSLCAD;
		}
		if (tref > 0.0 && tref < 60.0)
		  {
		  f1up= (AMPSLST-kfbasraf)*(1.0-Math.exp(-(tref)/taufras));
		  if (tref > 15.0) {f1dn=Math.exp(-(tref-15.0)/tausras);}
		  }
		if (tref > 0.0 && tref < 60.0)
		  {
		  g1up= (AMPSLSTD-kfbasrafd)*(1.0-Math.exp(-(tref)/taufras));
		  if (tref > 15.0) {g1dn=Math.exp(-(tref-15.0)/tausras);}
		  }
*/
// WLFS STIMULUS

		if ((tref > 0.0) && (tref < 15.0))
		{
			 casyn=AMPWLCA;
			 cadend=AMPWLCAD;
		}
		if (tref > 0.0 && tref < 60.0)
		  {
		  f1up= (AMPWLST-kfbasraf)*(1.0-Math.exp(-(tref)/taufras));
		  if (tref > 15.0) {f1dn=Math.exp(-(tref-15.0)/tausras);}
		  }
		if (tref > 0.0 && tref < 60.0)
		  {
		  g1up= (AMPWLSTD-kfbasrafd)*(1.0-Math.exp(-(tref)/taufras));
		  if (tref > 15.0) {g1dn=Math.exp(-(tref-15.0)/tausras);}
		  }


/* NOW REPEAT these protocols for STC or SCTC simulations in which synapse 2 activates the dendrite. */

	tref2 = tref-delt; // Offsets PRP stimulus to be del minutes later than Tag stimulus at synapse 1.

// Tetanic protocol
/*
		if (tref2 > 0.0 && tref2 < 0.05)
		  {
		  cadend=AMPTETCAD;
		  }
		if (tref2 > (0.0+isi) && tref2 < (0.0+isi+0.05))
		  {
		  cadend=AMPTETCAD;
		  }
		if (tref2 > (0.0+2.0*isi) && tref2 < (0.0+2.0*isi+0.05))
		  {
		  cadend=AMPTETCAD;
		  }
		if (tref2 > 0.0 && tref2 < 60.0)
		  {
		  g4up = 1.0-Math.exp(-(tref2)/taufras);
		  g4dn = (AMPTETSTIMD-kfbasrafd)* Math.exp(-(tref2)/tausras);
		  }
		if (tref2 > (0.0+isi) && tref2 < 60.0)
		  {
		  g5up = 1.0-Math.exp(-(tref2-isi)/taufras);
		  g5dn = (AMPTETSTIMD-kfbasrafd)* Math.exp(-(tref2-isi)/tausras);
		  }
		if (tref2 > (0.0+2.0*isi) && tref2 < 60.0)
		  {
		  g6up = 1.0-Math.exp(-(tref2-2.0*isi)/taufras);
		  g6dn = (AMPTETSTIMD-kfbasrafd)* Math.exp(-(tref2-2.0*isi)/tausras);
		  }
*/

// SLFS STIMULUS TO PRODUCE L-LTD

		if (tref2 > 0.0 && tref2 < 15.0 && cadend < AMPSLCAD)
		{
			 cadend=AMPSLCAD;
		}
		if (tref2 > 0.0 && tref2 < 60.0)
		  {
	 g4up= (AMPSLSTD-kfbasrafd)*(1.0-Math.exp(-(tref2)/taufras));
		  if (tref2 > 15.0) {g4dn=Math.exp(-(tref2-15.0)/tausras);}
		  }


// GIVE COMPOSITE KFRAFS
		if (tref > -300.0 && tref < 300.0)
		  {
kfraf=kfbasraf + (f1up*f1dn+f2up*f2dn+f3up*f3dn);
kfrafd=kfbasrafd + (g1up*g1dn+g2up*g2dn+g3up*g3dn) + (g4up*g4dn+g5up*g5dn+g6up*g6dn);
		  }


// INHIBITOR APPLICATIONS. FIRST SET THE BASELINE NONINHIBITED FACTOR VALUES
// TO 1.

		inherk=1.0;
		inhck2=1.0;
		inhprot=1.0;
		inhmkk=1.0;
		inhpp=1.0;
		inhpkm=1.0;

		if (tref > 300.0 && tref < 360.0)
		  {

/* ERK inhibition, as of March 14, blocks both LLTD and LLTP, as it should. */
//			inherk=0.1; 

//			inhck2=0.1;

//			inhprot=0.25; 

//			inhmkk=0.1; 

//			inhpp=0.1; 

//			inhpkm=0.2;
		  }

// BEGINNING OF ACTUAL CODE FOR UPDATING DYNAMIC VARIABLES. 

// DEFINE SOME AUXILIARY FUNCTIONS
		powca=casyn*casyn*casyn*casyn;
		powcad=cadend*cadend*cadend*cadend;

		powkc=Kck2*Kck2*Kck2*Kck2;
		powkcd=Kck2d*Kck2d*Kck2d*Kck2d;
		powkc2=Kpp*Kpp*Kpp*Kpp;

		rafp=raftot-raf;
		mkkp=mkktot-mkk-mkkpp;
		erkp=erktot-erk-erkpp;

		rafp2=raftot-raf2;
		mkkp2=mkktot-mkk2-mkkpp2;
		erkp2=erktot-erk2-erkpp2;

// Activation of CAMKII by a power of synaptic calcium, hill function,
// uses fourth powers.

		dv1dt = kfck2*(powca/(powca+powkc)) - ck2act/tauck2;

		dv2dt = erksmult*(-kfraf*raf+kbraf*rafp);

		dv3dt = erksmult*(-inhmkk*kfmkk*rafp*mkk/(mkk+Kmkk)+kbmkk*mkkp/(mkkp+Kmkk)); 

		dv4dt = erksmult*(inhmkk*kfmkk*rafp*mkkp/(mkkp+Kmkk)-kbmkk*mkkpp/(mkkpp+Kmkk));

		dv5dt = erksmult*(-kferk*mkkpp*erk/(erk+Kmk)
			+kberk*erkp/(erkp+Kmk));

		dv6dt = erksmult*(kferk*mkkpp*erkp/(erkp+Kmk)
			 -kberk*erkpp/(erkpp+Kmk));

		erkact = erkpp;

// ODE for LTD phosphatase activation

		dv7dt = kfpp*(powca/(powca+powkc2))-ppact/taupp;

// ODEs for tag phosphorylation, tag is at synapse 1

		dv8dt = kphos1*inhck2*ck2act*(1.0-tagp1) - kdeph1*tagp1;

/* Drive PRP synthesis by a different erk and camk activity. That is an easier way to simulate crosstagging at two synapses, without having to duplicate all the variables. I still have to duplicate everything down to ERK activation. */

// ODEs for Depression Tag Phospho Sites

		dv9dt = inherk*kphos4*erkact*(1.0-tagd1)-kdeph4*tagd1;

		dv10dt = inhpp*kdeph5*ppact*(1.0-tagd2)-kphos5*tagd2;

/* Synapse 1 is always considered as the tagged synapse, synapse 2 the prp synapse */

		tltp = tagp1*tagp1;
		tltd = tagd1*tagd2;

/* Important note – Feb 24 2011. I removed ERK from LTP TAG, from tltp. That is, the site tagp3 is not being used now! Because Sajikumar and Frey 2007 say ERK is NOT needed for setting the LTP tag. Instead it is needed for PRP synthesis. */

/* ODEs for dendrite. */

		dv11dt = kfck2*(powcad/(powcad+powkcd)) - ckdact/tauck2;

		dv12dt = erkdmult*(-kfrafd*raf2+kbraf*rafp2);

		dv13dt = erkdmult*(-inhmkk*kfmkk*rafp2*mkk2/(mkk2+Kmkk)+kbmkk*mkkp2/(mkkp2+Kmkk));

		dv14dt = erkdmult*(inhmkk*kfmkk*rafp2*mkkp2/(mkkp2+Kmkk)-kbmkk*mkkpp2/(mkkpp2+Kmkk));

		dv15dt = erkdmult*(-kferk*mkkpp2*erk2/(erk2+Kmk)
			+kberk*erkp2/(erkp2+Kmk));

		dv16dt = erkdmult*(kferk*mkkpp2*erkp2/(erkp2+Kmk)
			 -kberk*erkpp2/(erkpp2+Kmk));

		erkdact = erkpp2;

// ODE for a dendritic ERK phosphor site.

		dv17dt = inherk*kphos7*erkdact*(1.0-psit1)-kdeph7*psit1;

// ODE for phosphorylation by camkii of site that governs PKM synthesis

		dv18dt = inhck2*kphos8*ckdact*(1.0-psit2)-kdeph8*psit2;

/* Following ODE is synthesis of a PRP that is needed for LTD */

		dv19dt = inhprot*ktrans1*psit1*psit1-prp/tauprp + inhprot*vtrbas1;

/* ODE for dendritic PKM */

		dv20dt = pkmmult*(inhprot*kpkmon*psit1*psit2-pkmd/taupkm + inhprot*vtrbaspkm-kexpkm*tltp*pkmd+Vsd*klkpkm*pkmsn);

/* synaptic PKM */

dv21dt = kexpkm*tltp*pkmd/Vsd-klkpkm*pkmsn + inhprot*vtrbaspkm + kpkmon2*inhpkm*inhpkm*inhprot*pkmsn*pkmsn/(inhpkm*inhpkm*pkmsn*pkmsn+Kpkm*Kpkm)-pkmsn/taupkm;

// Fsyn ODE
		dv22dt = kltp*inhpkm*pkmsn-fsyn/tfsyn + vfbas; 

// nsyn ODE
		dv23dt = -kltd*tltd*prp*nsyn-nsyn/tnsyn + vdbas; 

		wsyn=fsyn*nsyn;
		values[24]=fsyn*nsyn;


		values[1]+=dt*dv1dt;
		values[2]+=dt*dv2dt;
		values[3]+=dt*dv3dt;
		values[4]+=dt*dv4dt;
		values[5]+=dt*dv5dt;
		values[6]+=dt*dv6dt;
		values[7]+=dt*dv7dt;
		values[8]+=dt*dv8dt;
		values[9]+=dt*dv9dt;
		values[10]+=dt*dv10dt;
		values[11]+=dt*dv11dt;
		values[12]+=dt*dv12dt;
		values[13]+=dt*dv13dt;
		values[14]+=dt*dv14dt;
		values[15]+=dt*dv15dt;
		values[16]+=dt*dv16dt;
		values[17]+=dt*dv17dt;
		values[18]+=dt*dv18dt;
		values[19]+=dt*dv19dt;
		values[20]+=dt*dv20dt;
		values[21]+=dt*dv21dt;
		values[22]+=dt*dv22dt;
		values[23]+=dt*dv23dt;

/* Equilibrate basal synaptic weight, prior to stimulation, according to basal kinase activities. */

		if ((tref < -1.0) && (tref2 < -1.0))
		  {
       fsyn = (kltp*pkmsn+vfbas)/(kltp*pkmsn+1.0/tfsyn);
       values[22] = (kltp*pkmsn+vfbas)/(kltp*pkmsn+1.0/tfsyn);
       
       nsyn = vdbas/(kltd*tltd*prp+1.0/tnsyn);
       values[23] = vdbas/(kltd*tltd*prp+1.0/tnsyn);

		  wsyn = fsyn*nsyn;
		  values[24] = fsyn*nsyn;
		  }

// Increment time
                time=time+dt;

// END INNER LOOP

                j++;
               } while (j <= delta/dt);

            k++;
           } while (k <= recend/delta);

		wend=wsyn;

			out2.println(delt + "\t" + wend);

            mtwo++;
           } while (mtwo <= 120);

// END OF LOOPS AND OF SIMULATION. CLOSE OUTPUT FILES.

      out2.close();

       }
}


import java.io.*;

public class stochpkm

/* Program for stochastic simulations in the top two panels of Figure 9 in the PKM manuscript. Uses Gillespie algorithm, uses single rate expression for the Hill-type function that governs the feedback-drive rate of PKM synthesis. */

 {  public static void main (String args[]) throws IOException {

    PrintWriter out2 = new PrintWriter(new FileWriter("avpkm.txt"));
    PrintWriter out5 = new PrintWriter(new FileWriter("pkmhi.txt"));
    PrintWriter out6 = new PrintWriter(new FileWriter("pkmlo.txt"));
    PrintWriter out7 = new PrintWriter(new FileWriter("tout.txt"));
    PrintWriter out8 = new PrintWriter(new FileWriter("stfig.txt"));

    int recintvl=300; // interval of steps to record, e.g. every 300th step

/* The following factor is needed to adjust molecule numbers for the volume of the simulated spine. Using parameters as in the deterministic ODE model, going from uM concentration to numbers in a spine volume, calculations give a value of 120 as appropriate for a large spine. */
	double stmult=120.0; // 120 for 0.2 um3 volume

	int rectime=10; /* How often to output data. Adjust depending on aknt and recend */

    	double recstart=0.01; // Time to start writing data
	double recend=recstart+144.0*60.0; // Time to end simulation. Time units are minutes, simulation of Fig. 9 is ended at 144 hrs.

	double tstim=recstart+72.0*60.0; // Time to apply stim
	double timewrite;

	double pkm; // pkm is the only dynamic variable

/* The stochastic simulation is repeated 20 times to allow for defining an average PKM trajectory as well as standard deviations around that average. */
	double avpkm;

/* Model parameters. As in ODE model except for stmult. */
	double kfpkm=0.055*stmult;
	double Kpkm=0.75*stmult;
	double kex=0.012;
	double vpkm=0.0003*stmult;
	double kdpkm=(1.0/50.0);

	double frac; // frac is the feedback Hill function

// Following quantities needed for Gillespie algorithm
	double rnum1;
	double rnum2;
	double rint1;
	double rint2;
	double ppi = 3.14159265;

	double trx;
	double asum;
	double rrx;
	int mu;

// standard deviation of PKM trajectories
	double stdev;

	double time; // time (seconds)

    int i, k, j, l, m, iout, aknt, aflip; // counters

	int[] nmols = new int[2];

	double[] au = new double[6];
	double[] as = new double[6];

	double[] pkmav = new double[3010];
	double[] pkm1 = new double[3010]; // Each of 20 simulations has its own timecourse array
	double[] pkm2 = new double[3010];
	double[] pkm3 = new double[3010];
	double[] pkm4 = new double[3010];
	double[] pkm5 = new double[3010];
	double[] pkm6 = new double[3010];
	double[] pkm7 = new double[3010];
	double[] pkm8 = new double[3010];
	double[] pkm9 = new double[3010];
	double[] pkm10 = new double[3010];
	double[] pkm11 = new double[3010];
	double[] pkm12 = new double[3010];
	double[] pkm13 = new double[3010];
	double[] pkm14 = new double[3010];
	double[] pkm15 = new double[3010];
	double[] pkm16 = new double[3010];
	double[] pkm17 = new double[3010];
	double[] pkm18 = new double[3010];
	double[] pkm19 = new double[3010];
	double[] pkm20 = new double[3010];
	double[] dev = new double[3010];
	double[] pkmh = new double[3010];
	double[] pkml = new double[3010];
	double[] tout = new double[3010];

        i=1;
        do { 
		pkmav[i]=0.0;
		pkmh[i]=0.0;
		pkml[i]=0.0;
		dev[i]=0.0;
		pkm1[i]=0.0;
		pkm2[i]=0.0;
		pkm3[i]=0.0;
		pkm4[i]=0.0;
		pkm5[i]=0.0;
		pkm6[i]=0.0;
		pkm7[i]=0.0;
		pkm8[i]=0.0;
		pkm9[i]=0.0;
		pkm10[i]=0.0;
		pkm11[i]=0.0;
		pkm12[i]=0.0;
		pkm13[i]=0.0;
		pkm14[i]=0.0;
		pkm15[i]=0.0;
		pkm16[i]=0.0;
		pkm17[i]=0.0;
		pkm18[i]=0.0;
		pkm19[i]=0.0;
		pkm20[i]=0.0;
		tout[i]=0.0;
	   i++;
         } while (i <= 3007);

/* Following outer loop, with iout, runs over the 20 realizations of the PKM trajectories */
 
   iout=1;
    do { 

	time = 0.0;
	nmols[1]=(int)(vpkm/(kex+kdpkm)); // set PKM to basal level

	aknt=1;
	aflip=0;


/* Following inner loop goes over variable Gillespie timesteps, until either the output time of 144 hrs (for Fig 9) is reached or the pkm time course arrays come close to filling up. If the latter, rerun with larger arrays to reach target time. The loop counter is the variable aknt. Aknt also sweeps through and fills up the arrays. */

      do {  

	if ((time > tstim) && (aflip == 0))
	{

// Reset PKM to stimulated level at time tstim. Aflip makes sure it is only reset once.

      nmols[1]=(int)(1.4*stmult); 
	aflip=1;
	} 

	pkm=(double)(nmols[1]); 

// deterministic rate expressions needed for Gillespie algorithm

	frac = (pkm*pkm)/(pkm*pkm + Kpkm*Kpkm);
	au[1]=kfpkm*frac;
	au[2]=kex*pkm;
	au[3]=vpkm;
	au[4]=kdpkm*pkm;

// Following is gillespie code – generic for this algorithm

		asum = au[1];
		as[1] = au[1];

        j=2;
        do { 
 		asum = asum+au[j];
		as[j]= as[j-1]+au[j];
        j++;
        } while (j <= 4);

		rnum1=Math.random();
		rnum2=Math.random();

		if (rnum1 < 0.00000001)
		{
	  	rnum1 = 0.00000001;
		}
		trx=1.0/asum*Math.log(1.0/rnum1);
		time=time+trx;

		rrx=asum*rnum2;

		mu=5;

       	k=2;
       do { 
		if ((rrx < as[k]) && (rrx > as[k-1]))
		{
	  	mu=k;
		}
        	k++;
          } while (k <= 4);
		if (rrx < as[1])
		{
	  	mu=1;
		}

		if (mu == 1)
		{
		nmols[1]=nmols[1]+1;
		}

		if (mu == 2)
		{
		nmols[1]=nmols[1]-1;
		}

		if (mu == 3)
		{
		nmols[1]=nmols[1]+1;
		}

		if (mu == 4)
		{
		nmols[1]=nmols[1]-1;
		}

		if (nmols[1] < 0) {nmols[1] = 0;}

/* Following long, nested IF statements just fill up appropriate PKM timecourse arrays and also compute and add to the PKM average timecourse. Also, the array tout is used to hold an approximate, “average” simulation time variable. That time variable is used in plotting the graphs in Figure 9. That time variable is not perfect in that it can change abruptly when individual simulations out of the 20 end early, but it seems to me the best that can be done. Because of these changes, do the following. When output is complete, eliminate all tout values below the first time the targeted output time, 144 for Fig 9, is reached. Then plot the PKM values corresponding to the remaining output times. */

         if (time > ((double)(aknt*rectime)))
		{
	
		pkmav[aknt]=pkmav[aknt]+pkm/20.0;
		tout[aknt]=tout[aknt]+time/(60.0*20.0); // time in hrs here

		if (iout == 1)
		  {
   		    pkm1[aknt]=pkm;
		  }
		if (iout == 2)
		  {
   		    pkm2[aknt]=pkm;
		  }
		if (iout == 3)
		  {
   		    pkm3[aknt]=pkm;
		  }
		if (iout == 4)
		  {
   		    pkm4[aknt]=pkm;
		  }
		if (iout == 5)
		  {
   		    pkm5[aknt]=pkm;
		  }
		if (iout == 6)
		  {
   		    pkm6[aknt]=pkm;
		  }
		if (iout == 7)
		  {
   		    pkm7[aknt]=pkm;
		  }
		if (iout == 8)
		  {
   		    pkm8[aknt]=pkm;
		  }
		if (iout == 9)
		  {
   		    pkm9[aknt]=pkm;
		  }
		if (iout == 10)
		  {
   		    pkm10[aknt]=pkm;
		  }
		if (iout == 11)
		  {
   		    pkm11[aknt]=pkm;
		  }
		if (iout == 12)
		  {
   		    pkm12[aknt]=pkm;
		  }
		if (iout == 13)
		  {
   		    pkm13[aknt]=pkm;
		  }
		if (iout == 14)
		  {
   		    pkm14[aknt]=pkm;
		  }
		if (iout == 15)
		  {
   		    pkm15[aknt]=pkm;
		  }
		if (iout == 16)
		  {
   		    pkm16[aknt]=pkm;
		  }
		if (iout == 17)
		  {
   		    pkm17[aknt]=pkm;
		  }
		if (iout == 18)
		  {
   		    pkm18[aknt]=pkm;
		  }
		if (iout == 19)
		  {
   		    pkm19[aknt]=pkm;
		  }
		if (iout == 20)
		  {
   		    pkm20[aknt]=pkm;
		  }

		 aknt=aknt+1; // increment array counter

		}

// REPEAT OVER INNER LOOP, THEN OVER OUTER LOOP

           } while ((time < (1.5*recend)) && (aknt < 3000));


	   iout++;
         } while (iout <= 20);

// Simulation done, now output data. Loop over PKM array elements.

        i=1;
        do { 

		 timewrite=tout[i];
            out2.println(timewrite + "\t" + pkmav[i]); // Averaged PKM time course over 20 simulations

/* This cumbersome code computes the standard deviation of the PKM trajectories around the average PKM trajectory. There may be a quicker way but this is simple. */
		dev[i]=(pkm1[i]-pkmav[i])*(pkm1[i]-pkmav[i]);
		dev[i]=dev[i]+(pkm2[i]-pkmav[i])*(pkm2[i]-pkmav[i]);
		dev[i]=dev[i]+(pkm3[i]-pkmav[i])*(pkm3[i]-pkmav[i]);
		dev[i]=dev[i]+(pkm4[i]-pkmav[i])*(pkm4[i]-pkmav[i]);
		dev[i]=dev[i]+(pkm5[i]-pkmav[i])*(pkm5[i]-pkmav[i]);
		dev[i]=dev[i]+(pkm6[i]-pkmav[i])*(pkm6[i]-pkmav[i]);
		dev[i]=dev[i]+(pkm7[i]-pkmav[i])*(pkm7[i]-pkmav[i]);
		dev[i]=dev[i]+(pkm8[i]-pkmav[i])*(pkm8[i]-pkmav[i]);
		dev[i]=dev[i]+(pkm9[i]-pkmav[i])*(pkm9[i]-pkmav[i]);
		dev[i]=dev[i]+(pkm10[i]-pkmav[i])*(pkm10[i]-pkmav[i]);
		dev[i]=dev[i]+(pkm11[i]-pkmav[i])*(pkm11[i]-pkmav[i]);
		dev[i]=dev[i]+(pkm12[i]-pkmav[i])*(pkm12[i]-pkmav[i]);
		dev[i]=dev[i]+(pkm13[i]-pkmav[i])*(pkm13[i]-pkmav[i]);
		dev[i]=dev[i]+(pkm14[i]-pkmav[i])*(pkm14[i]-pkmav[i]);
		dev[i]=dev[i]+(pkm15[i]-pkmav[i])*(pkm15[i]-pkmav[i]);
		dev[i]=dev[i]+(pkm16[i]-pkmav[i])*(pkm16[i]-pkmav[i]);
		dev[i]=dev[i]+(pkm17[i]-pkmav[i])*(pkm17[i]-pkmav[i]);
		dev[i]=dev[i]+(pkm18[i]-pkmav[i])*(pkm18[i]-pkmav[i]);
		dev[i]=dev[i]+(pkm19[i]-pkmav[i])*(pkm19[i]-pkmav[i]);
		dev[i]=dev[i]+(pkm20[i]-pkmav[i])*(pkm20[i]-pkmav[i]);

		dev[i]=dev[i]/20.0;
		dev[i]=Math.sqrt(dev[i]);

/* Now fill two arrays with trajectories that are 1 standard dev. above and below the average trajectory */
		pkmh[i]=pkmav[i]+dev[i];
		pkml[i]=pkmav[i]-dev[i];
		if (pkml[i] < 0.0) 
       { 
       pkml[i]=0.0;
       }

/* Write hi and lo timecourses. File 8 has three timecourses in one file. */
		 out5.println(timewrite + "\t" + pkmh[i]);
		 out6.println(timewrite + "\t" + pkml[i]);
		 out7.println(tout[i]);
            out8.println(timewrite + "\t" + pkmav[i] + "\t" + pkmh[i] + "\t" + pkml[i]);


	   i++;
         } while (i <= (aknt-1));

      out2.close();
      out5.close();
      out6.close();
      out7.close();
      out8.close();

       }
}


import java.io.*;

public class elemstoch

/* This stochastic Gillespie simulation is for the lower panel in Figure 9. Here, elementary steps of PKM sequentially binding to a target, followed by PKM synthesis when the target is fully occupied by two PKMs, replace the composite Hill rate function for the rate of PKM synthesis due to feedback that was used in stochpkm.java. */

 {  public static void main (String args[]) throws IOException {

    PrintWriter out1 = new PrintWriter(new FileWriter("pkmone.txt"));
    PrintWriter out2 = new PrintWriter(new FileWriter("avpkm.txt"));
    PrintWriter out5 = new PrintWriter(new FileWriter("pkmhi.txt"));
    PrintWriter out6 = new PrintWriter(new FileWriter("pkmlo.txt"));
    PrintWriter out7 = new PrintWriter(new FileWriter("tout.txt"));
    PrintWriter out8 = new PrintWriter(new FileWriter("stfig.txt"));
    PrintWriter out9 = new PrintWriter(new FileWriter("t1one.txt"));
    PrintWriter out10 = new PrintWriter(new FileWriter("t2one.txt"));

    int recintvl=600; // intervals of steps to record

	double stmult=120.0; // 120 for 0.2 um3 volume

	int rectime=1; // depends on aknt and recend

    	double recstart=0.0; // Time to start writing data

	double tstim=72.0*60.0; // Time to apply stim
	double recend=144.0*60.0; // Time to end simulation

	double timewrite;

	double pkm;
	double avpkm;

	double ttot=1.5*stmult;
/* Ttot is the target for PKM sequential binding. When it is twice bound, it causes synthesis of PKM. Positive feedback will be more effective if Ttot is present in excess over the maximum of PKM (the upper state of a bistable PKM switch). Other parameters are qualitatively same as in the previous stochastic simulation (stochpkm.java, top two panels of Fig. 9). For the elementary step simulation, the forward and backward binding rate constants of PKM to ttot are chosen to have a ratio compatible to the Kpkm dissociation constant of PKM in stochpkm.java. Their absolute values are chosen such that binding is the fastest process, but not that much faster than other processes (so that simul does not take too long). */

	double t1; // t1 and t2 are states of Ttot that have one or two PKMs bound.
	double t2;

// Remainder of code is very similar to stochpkm.java, and will not be commented on in as much detail.

	double kfpkm=0.055;
	double Kpkm=0.75*stmult;
	double kf=10.0/stmult; // Forward and backward binding rates in accordance with Kpkm from stochpkm.java
	double kb=Kpkm*kf;
	double kex=0.012;
	double vpkm=0.0003*stmult;
	double kdpkm=(1.0/50.0);

	double rnum1;
	double rnum2;
	double rint1;
	double rint2;
	double ppi = 3.14159265;

	double trx;
	double asum;
	double rrx;
	int mu;

	double stdev;

	double time; // time (seconds)

    int i, k, j, l, m, iout, aknt, aflip; // counters

	int[] nmols = new int[5];

	double[] au = new double[10];
	double[] as = new double[10];

	double[] pkmav = new double[15010];
	double[] pkm1 = new double[15010];
	double[] t1arr = new double[15010];
	double[] t2arr = new double[15010];
	double[] pkm2 = new double[15010];
	double[] pkm3 = new double[15010];
	double[] pkm4 = new double[15010];
	double[] pkm5 = new double[15010];
	double[] pkm6 = new double[15010];
	double[] pkm7 = new double[15010];
	double[] pkm8 = new double[15010];
	double[] pkm9 = new double[15010];
	double[] pkm10 = new double[15010];
	double[] pkm11 = new double[15010];
	double[] pkm12 = new double[15010];
	double[] pkm13 = new double[15010];
	double[] pkm14 = new double[15010];
	double[] pkm15 = new double[15010];
	double[] pkm16 = new double[15010];
	double[] pkm17 = new double[15010];
	double[] pkm18 = new double[15010];
	double[] pkm19 = new double[15010];
	double[] pkm20 = new double[15010];
	double[] dev = new double[15010];
	double[] pkmh = new double[15010];
	double[] pkml = new double[15010];
	double[] tout = new double[15010];

        i=1;
        do { 
		pkmav[i]=0.0;
		pkmh[i]=0.0;
		pkml[i]=0.0;
		dev[i]=0.0;
		pkm1[i]=0.0;
		t1arr[i]=0.0;
		t2arr[i]=0.0;
		pkm2[i]=0.0;
		pkm3[i]=0.0;
		pkm4[i]=0.0;
		pkm5[i]=0.0;
		pkm6[i]=0.0;
		pkm7[i]=0.0;
		pkm8[i]=0.0;
		pkm9[i]=0.0;
		pkm10[i]=0.0;
		pkm11[i]=0.0;
		pkm12[i]=0.0;
		pkm13[i]=0.0;
		pkm14[i]=0.0;
		pkm15[i]=0.0;
		pkm16[i]=0.0;
		pkm17[i]=0.0;
		pkm18[i]=0.0;
		pkm19[i]=0.0;
		pkm20[i]=0.0;
		tout[i]=0.0;
	   i++;
         } while (i <= 15007);

    iout=1;
    do { 

	time = 0.0;
	nmols[1]=(int)(vpkm/(kex+kdpkm)); // Basal PKM set as in stoch.java, basal Ttot state is with no PKM bound.
	nmols[2]=0;
	nmols[3]=0;

	aknt=1;
	aflip=0;

      do {  

	if ((time > tstim) && (aflip == 0))
	{
	nmols[1]=(int)(1.2*stmult);
	aflip=1;
	} 

	pkm=(double)(nmols[1]);
	t1=(double)(nmols[2]);
	t2=(double)(nmols[3]);

/* Following deterministic rates differ from stochpkm.java in that a couple extra steps are added to describe sequential binding. These rates are au[5-8]. Note that ttot-t1-t2 is the amount of target with no PKM bound. */

/* au[1], the rate of PKM synthesis due to positive feedback, is now proportional to the amount of target occupied by two PKMs. */

	au[1]=kfpkm*t2;
	au[2]=kex*pkm;
	au[3]=vpkm;
	au[4]=kdpkm*pkm;

	au[5]=kf*pkm*(ttot-t1-t2);
	if (au[5] < 0.0) {au[5] = 0.0;}
	au[6]=kb*t1;
	au[7]=kf*pkm*t1;
	au[8]=kb*t2;

		asum = au[1];
		as[1] = au[1];

        j=2;
        do { 
 		asum = asum+au[j];
		as[j]= as[j-1]+au[j];
        j++;
        } while (j <= 8);

		rnum1=Math.random();
		rnum2=Math.random();

		if (rnum1 < 0.00000001)
		{
	  	rnum1 = 0.00000001;
		}
		trx=1.0/asum*Math.log(1.0/rnum1);
		time=time+trx;

		rrx=asum*rnum2;

		mu=9;

       	k=2;
       do { 
		if ((rrx < as[k]) && (rrx > as[k-1]))
		{
	  	mu=k;
		}
        	k++;
          } while (k <= 8);
		if (rrx < as[1])
		{
	  	mu=1;
		}

		if (mu == 1)
		{
		nmols[1]=nmols[1]+1;
		}

		if (mu == 2)
		{
		nmols[1]=nmols[1]-1;
		}

		if (mu == 3)
		{
		nmols[1]=nmols[1]+1;
		}

		if (mu == 4)
		{
		nmols[1]=nmols[1]-1;
		}
		if (mu == 5)
		{
		nmols[2]=nmols[2]+1;
		}
		if (mu == 6)
		{
		nmols[2]=nmols[2]-1;
		}
		if (mu == 7)
		{
		nmols[3]=nmols[3]+1;
		nmols[2]=nmols[2]-1;
		}
		if (mu == 8)
		{
		nmols[3]=nmols[3]-1;
		nmols[2]=nmols[2]+1;
		}

		if (nmols[1] < 0) {nmols[1] = 0;}
		if (nmols[2] < 0) {nmols[2] = 0;}
		if (nmols[3] < 0) {nmols[3] = 0;}

         if (time > ((double)(aknt*rectime)))
		{
	
		pkmav[aknt]=pkmav[aknt]+pkm/20.0;
		tout[aknt]=tout[aknt]+time/(60.0*20.0); // time in hrs here

		if (iout == 1)
		  {
   		    pkm1[aknt]=pkm;
   		    t1arr[aknt]=t1;
   		    t2arr[aknt]=t2;
		  }
		if (iout == 2)
		  {
   		    pkm2[aknt]=pkm;
		  }
		if (iout == 3)
		  {
   		    pkm3[aknt]=pkm;
		  }
		if (iout == 4)
		  {
   		    pkm4[aknt]=pkm;
		  }
		if (iout == 5)
		  {
   		    pkm5[aknt]=pkm;
		  }
		if (iout == 6)
		  {
   		    pkm6[aknt]=pkm;
		  }
		if (iout == 7)
		  {
   		    pkm7[aknt]=pkm;
		  }
		if (iout == 8)
		  {
   		    pkm8[aknt]=pkm;
		  }
		if (iout == 9)
		  {
   		    pkm9[aknt]=pkm;
		  }
		if (iout == 10)
		  {
   		    pkm10[aknt]=pkm;
		  }
		if (iout == 11)
		  {
   		    pkm11[aknt]=pkm;
		  }
		if (iout == 12)
		  {
   		    pkm12[aknt]=pkm;
		  }
		if (iout == 13)
		  {
   		    pkm13[aknt]=pkm;
		  }
		if (iout == 14)
		  {
   		    pkm14[aknt]=pkm;
		  }
		if (iout == 15)
		  {
   		    pkm15[aknt]=pkm;
		  }
		if (iout == 16)
		  {
   		    pkm16[aknt]=pkm;
		  }
		if (iout == 17)
		  {
   		    pkm17[aknt]=pkm;
		  }
		if (iout == 18)
		  {
   		    pkm18[aknt]=pkm;
		  }
		if (iout == 19)
		  {
   		    pkm19[aknt]=pkm;
		  }
		if (iout == 20)
		  {
   		    pkm20[aknt]=pkm;
		  }

		 aknt=aknt+1;

		}

           } while ((time < (1.01*recend)) && (aknt < 20000));

// END OUTER LOOP, REPEAT

	   iout++;
         } while (iout <= 20);

        i=1;
        do { 

		 timewrite=tout[i]/24.0;
            out1.println(timewrite + "\t" + pkm1[i]);
            out9.println(timewrite + "\t" + t1arr[i]);
            out10.println(timewrite + "\t" + t2arr[i]);
            out2.println(timewrite + "\t" + pkmav[i]);

		dev[i]=(pkm1[i]-pkmav[i])*(pkm1[i]-pkmav[i]);
		dev[i]=dev[i]+(pkm2[i]-pkmav[i])*(pkm2[i]-pkmav[i]);
		dev[i]=dev[i]+(pkm3[i]-pkmav[i])*(pkm3[i]-pkmav[i]);
		dev[i]=dev[i]+(pkm4[i]-pkmav[i])*(pkm4[i]-pkmav[i]);
		dev[i]=dev[i]+(pkm5[i]-pkmav[i])*(pkm5[i]-pkmav[i]);
		dev[i]=dev[i]+(pkm6[i]-pkmav[i])*(pkm6[i]-pkmav[i]);
		dev[i]=dev[i]+(pkm7[i]-pkmav[i])*(pkm7[i]-pkmav[i]);
		dev[i]=dev[i]+(pkm8[i]-pkmav[i])*(pkm8[i]-pkmav[i]);
		dev[i]=dev[i]+(pkm9[i]-pkmav[i])*(pkm9[i]-pkmav[i]);
		dev[i]=dev[i]+(pkm10[i]-pkmav[i])*(pkm10[i]-pkmav[i]);
		dev[i]=dev[i]+(pkm11[i]-pkmav[i])*(pkm11[i]-pkmav[i]);
		dev[i]=dev[i]+(pkm12[i]-pkmav[i])*(pkm12[i]-pkmav[i]);
		dev[i]=dev[i]+(pkm13[i]-pkmav[i])*(pkm13[i]-pkmav[i]);
		dev[i]=dev[i]+(pkm14[i]-pkmav[i])*(pkm14[i]-pkmav[i]);
		dev[i]=dev[i]+(pkm15[i]-pkmav[i])*(pkm15[i]-pkmav[i]);
		dev[i]=dev[i]+(pkm16[i]-pkmav[i])*(pkm16[i]-pkmav[i]);
		dev[i]=dev[i]+(pkm17[i]-pkmav[i])*(pkm17[i]-pkmav[i]);
		dev[i]=dev[i]+(pkm18[i]-pkmav[i])*(pkm18[i]-pkmav[i]);
		dev[i]=dev[i]+(pkm19[i]-pkmav[i])*(pkm19[i]-pkmav[i]);
		dev[i]=dev[i]+(pkm20[i]-pkmav[i])*(pkm20[i]-pkmav[i]);

		dev[i]=dev[i]/20.0;
		dev[i]=Math.sqrt(dev[i]);

		pkmh[i]=pkmav[i]+dev[i];
		pkml[i]=pkmav[i]-dev[i];
		if (pkml[i] < 0.0) 
       { 
       pkml[i]=0.0;
       }

		 out5.println(timewrite + "\t" + pkmh[i]);
		 out6.println(timewrite + "\t" + pkml[i]);
		 out7.println(tout[i]);
            out8.println(timewrite + "\t" + pkmav[i] + "\t" + timewrite + "\t" + pkmh[i] + "\t" + timewrite + "\t" + pkml[i]);


	   i++;
         } while (i <= (aknt-1));

      out1.close();
      out2.close();
      out5.close();
      out6.close();
      out7.close();
      out8.close();
      out9.close();
      out10.close();

       }
}
